# Evaluation of normalization strategies for mass spectrometry-based multi-omics datasets

**DOI:** 10.1007/s11306-025-02297-1

**Published:** 2025-07-01

**Authors:** Chi Yen Tseng, Jessica A. Salguero, Joshua D. Breidenbach, Emilia Solomon, Claire K. Sanders, Tara Harvey, M. Grace Thornhill, Salvator J. Palmisano, Zachary J. Sasiene, Brett R. Blackwell, Ethan M. McBride, Kes A. Luchini, Erick S. LeBrun, Marc Alvarez, Phillip M. Mach, Emilio S. Rivera, Trevor G. Glaros

**Affiliations:** 1 Biochemistry and Biotechnology Group, Bioscience Division, Los Alamos National Laboratory, Los Alamos, NM 84545 USA; 2 Microbial and Biome Sciences Group, Bioscience Division, Los Alamos National Laboratory, Los Alamos, NM 87545 USA; 3 LMI, Colorado Springs, CO 80919 USA

**Keywords:** Normalization, Multi-omics, Time-course, Metabolomics, Lipidomics, Proteomics

## Abstract

**Introduction:**

Data normalization is crucial for multi-omics integration, reducing systematic errors and maximizing the likelihood of discovering true biological variation. Most studies assess normalization for a single omics type or use datasets from separate experiments. Few address time-course data, where normalization might bias temporal differentiation. In this study, we compared common normalization methods and a machine learning approach, Systematical Error Removal using Random Forest (SERRF), using multi-omics datasets generated from the same experiment—even from the same cell lysate.

**Objectives:**

To develop a straightforward process to assess normalization effects and identify the most robust methods across multi-omics datasets.

**Methods:**

We analyzed metabolomics, lipidomics, and proteomics datasets from primary human cardiomyocytes and motor neurons exposed to acetylcholine-active compounds over time. Normalization effectiveness was evaluated based on improvement in QC features consistency and observing the change in treatment and time-related variance.

**Results:**

Probabilistic Quotient Normalization (PQN) and Locally Estimated Scatterplot Smoothing (LOESS) QC were identified as optimal for metabolomics and lipidomics, while PQN, Median, and LOESS normalization excelled for proteomics. These methods consistently enhanced QC feature consistency in metabolomics and lipidomics, and preserved time-related variance or treatment-related variance in proteomics, demonstrating their effectiveness and robustness. SERRF normalization, applied only to metabolomics in this study, outperformed other methods in some datasets but inadvertently masked treatment-related variance in others.

**Conclusion:**

Our evaluation identified PQN and LoessQC as the top methods for metabolomics and lipidomics, and PQN, Median, and Loess normalization for proteomics, in multi-omics integration in a temporal study.

**Supplementary Information:**

The online version contains supplementary material available at 10.1007/s11306-025-02297-1.

## Introduction

Data normalization is a critical preprocessing step in the analysis of mass spectrometry-based omics datasets, including metabolomics, proteomics, and lipidomics. Its primary purpose is to maximize the discovery of meaningful biological differences by reducing systematic technical variation arising from discrepancies in sample preparation, extraction, digestion, and instrumental noise, which are often uncontrollable in an experiment (Katajamaa and Orešič, 2007; Mertens, 2017). Despite its importance, many studies evaluating normalization methods often overlook how these methods alter the underlying data structure or remove biological variation (Chua et al., 2023). Typically, evaluations focus on improving feature consistency in quality control (QC) samples and enhancing differentiation across treatments (Chen et al., 2017; Wang et al., 2013). However, the performance of a normalization strategy depends on the data structure (Callister et al., 2006; Chen et al., 2017; Misra, 2020) and inappropriate normalization strategies can obscure genuine biological signals, leading to inaccurate findings (Chen et al., 2017; Cuevas-Delgado et al., 2020; Dubois et al., 2020; Wang et al., 2021). This issue is particularly evident when sophisticated algorithms, which make rigid assumptions about the nature of bias or involve machine learning, overfit the data, introduce bias, and misinterpret biological phenomena (Fan et al., 2019; Liebal et al., 2020; Välikangas et al., 2018).

Time-course exposure datasets present additional challenges because both time and treatment factors contribute to variance, and these datasets naturally exhibit time-dependent variations in the data structure (Hejblum et al., 2015; Le et al., 2015; Rubingh et al., 2009). Limited studies have systematically addressed how different normalization methods affect such datasets—specifically, which methods can reduce systematic variation without distorting the underlying longitudinal data structure (Sindelar et al., 2021). Avoiding normalization for a longitudinal study to keep pure biological responses has also been reported (Deda et al., 2017). We aim to identify a robust and effective normalization method by evaluating its impact on the variance in feature space attributable to time and treatment. Datasets should preserve time or treatment-related variance following normalization.

In addition to time-course challenges, most normalization evaluations for mass spectrometry-based omics focus on one or two types of omics data rather than including multiple omics platforms (Chua et al., 2023; Li et al., 2016; Välikangas et al., 2018). Each omics type possesses distinct characteristics that influence mass spectral identification (Smith et al., 2014) and quantification (Khoury et al., 2018). Well-known factors that affect omics data analysis include the variation in fragmentation, ionization efficiency, enzyme digestion rate, ion suppression, and spectral counting (Smith et al., 2014). These technical factors affect omics feature identification, quantification, and missing value generation, varying by omics type and complicate the selection of normalization methods (Brombacher et al., 2025; Flores et al., 2023; Kong et al., 2022).

Additionally, normalization methods differ depending on their underlying assumptions and the use of pooled QC samples, which are created by mixing small amounts of multiple individual samples from a study. These methods assume datasets have similar distribution or consistent trends to from which optimal scaling factors can be derived (Callister et al., 2006; Veselkov et al., 2011). Some advanced normalization approaches utilize pooled QC samples to learn feature correlations and address variation in sample injection order, e.g., Systematical Error Removal using Random Forest (SERRF) (Fan et al., 2019). However, it remains uncertain whether these assumptions or machine learning strategies perform robustly in multi-omics experiments or if a single normalization method can perform reliably across diverse omics datasets.

Comparing normalization methods for multi-omics data introduces several challenges. Omics data are often measured from separate experiments and samples, complicating the separation of biological variation from instrumental variations, experimental conditions, and individual sample differences (Conesa & Beck, 2019). A robust normalization method would perform equally well for omics datasets with higher or lower variation in total intensity (Välikangas et al., 2018; Zhao et al., 2020) and identifying such a method requires careful evaluation.

In this study, we address these challenges by systematically evaluating common normalization methods and machine learning-based SERRF normalization across metabolomics, proteomics, and lipidomics datasets derived from the same biological samples, even from the same lysate. Omics measurements were taken from primary human cardiomyocytes and motor neurons exposed to acetylcholine-active compounds to evaluate the performance of different normalization methods in datasets with higher variability in feature intensity across samples (cardiomyocytes) versus lower variability (neurons). To determine robust normalization strategies suitable for different omics datasets, we focus on examining how these methods affect variance explained by time and treatments while enhancing feature consistency in QC samples. Furthermore, we investigate how the inherent characteristics of each omics dataset impact the effectiveness of normalization methods. Our goal is to provide a framework that guides the selection of appropriate normalization techniques in multi-omics studies, ultimately improving data integration and the reliability of biological interpretations.

## Materials and methods

### Cell culture, exposure, lysis, sample preparation, and data acquisition

Human iPSC-derived motor neurons and commercially sourced cardiomyocytes were cultured and maintained as detailed in the recent publication (Rivera et al., 2024). Neurons and cardiomyocytes were exposed to carbaryl (Sigma Aldrich, product number 32055, CAS 63-25-2) and chlorpyrifos (Supelco, product number 45395, CAS 2921-882) at 0.1 µM with acetonitrile (ACN) as a vehicle control. Cells were collected at 5, 15, 30, 60, 120, 240, 480, 720, 1440 min post-exposure. Information about cell lysis, sample extraction, data collection for metabolomics, lipidomics, and proteomics is described in Supplementary Document 1 and in previously published work (Rivera et al., 2024). Metabolomics datasets were acquired using reverse-phase (RP) and hydrophilic interaction chromatography (HILIC) in both positive and negative ionization modes. Lipidomics datasets were acquired in positive and negative modes. Proteomics datasets were acquired using RP chromatography in positive mode. Detailed information regarding cell culture, exposure, lysis, sample preparation, and data acquisition is also available in recent work by our group (Breidenbach et al., 2024; Rivera et al., 2024). The multi-omics datasets were generated from the same samples and lysates.

### Data preparation

**Fig. 1:**
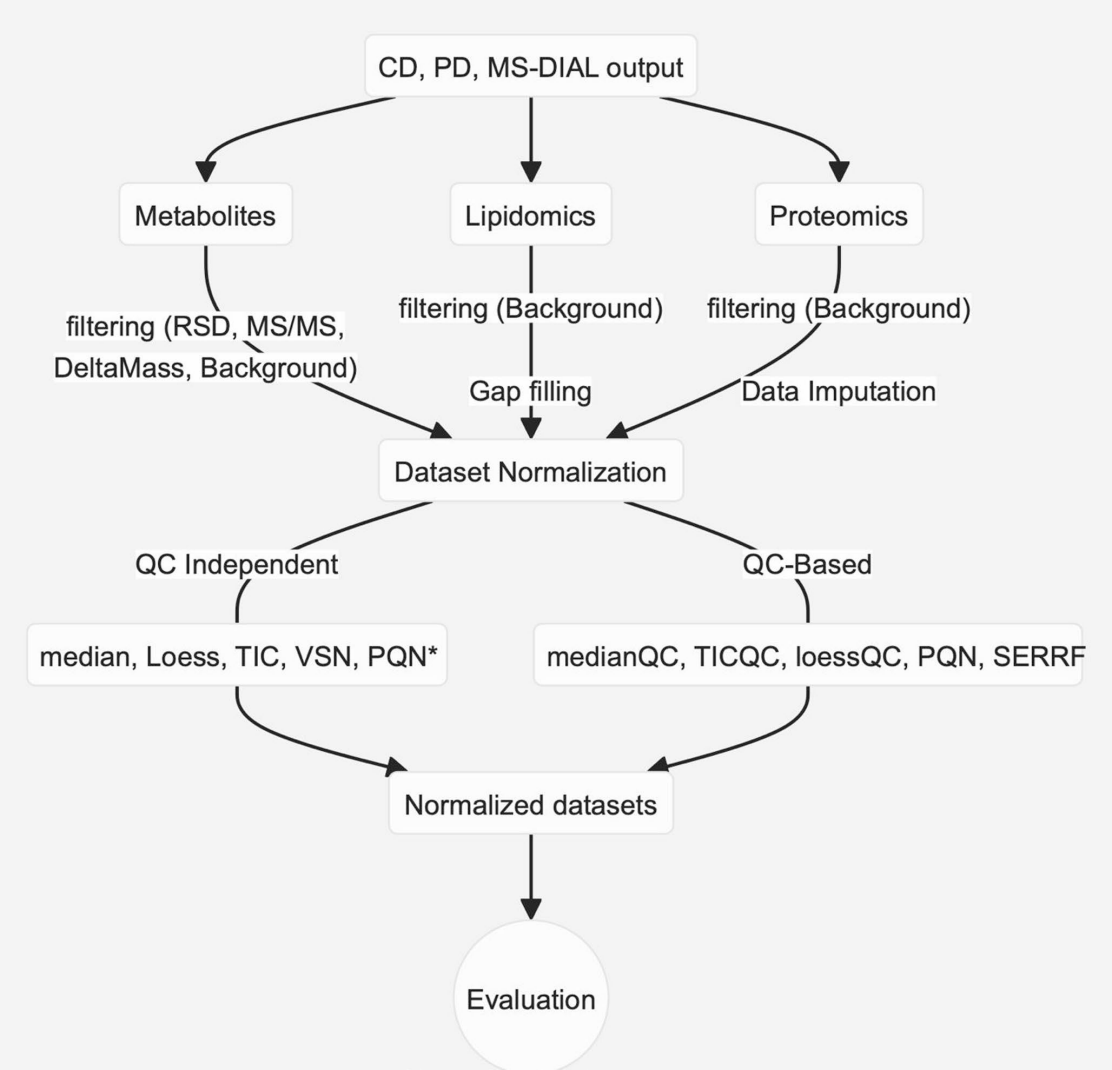
Multi-omics data were preprocessed by applying filtering, imputation, and normalization. Two distinct strategies were used: one based on the average sample as the reference (QC-independent) and another relying solely on pooled QC samples (QC-based). The resulting normalized datasets were evaluated to assess the effectiveness of each normalization approach

An overview of the multi-omics data pre-processing and normalization evaluation steps is shown in Fig. 1. We processed metabolomics datasets using Compound Discoverer 3.3 (Thermo Scientific, San Jose, CA), lipidomics datasets using MS-DIAL 5.1 (Tsugawa et al., 2020), and proteomics datasets using Proteome Discoverer 3.0 (Thermo Scientific, San Jose, CA). Additional details on data preprocessing, including filtering, missing value imputation and gap filling, are described in Supplementary Document 1.

### Normalization methods

Normalization methods were designed to either match the total intensity of all features and/or the distribution of feature intensities across all samples. We selected six commonly used normalization methods based on different assumptions. Total Ion Current (TIC) normalization assumes that total feature intensity is consistent across samples. Locally Estimated Scatterplot Smoothing (LOESS) normalization assumes balanced proportions of upregulated and downregulated features. Median normalization assumes constant median feature intensity across samples. Quantile normalization assumes overall distribution of feature intensities is similar and can be mapped to the same percentile of the normal distribution. LOESS, Median, and Quantile normalization were performed using the limma package in R. (Ritchie et al., 2015). Probabilistic Quotient Normalization (PQN) also assumes overall distribution of feature intensities is similar across samples; however, instead of assuming normal distribution like Quantile normalization, PQN adjusts the distribution based on the ranking of a reference spectrum. A reference spectrum (the median spectrum from either pooled QC samples or all the samples) is used for estimating dilution factors based on the relative ratio for PQN (Callister et al., 2006; Veselkov et al., 2011). Variance Stabilizing Normalization (VSN) assumes feature variances are dependent on their mean and a transformation can make variance approximately constant and comparable across features. VSN is only selected method that transforms the data distribution. VSN was applied using the vsn package in R (Huber et al., 2002) for proteomics only. TICQC, medianQC, and LOESSQC normalization methods were executed similarly to their standard counterparts, but with each sample normalized individually against all QC samples at a time. Machine learning-based SERRF normalization uses correlated compounds in QC samples to correct systematic errors, including batch effects and injection order. SERRF was applied to the datasets from Compound Discoverer 3.3 software (Thermo Scientific) (Fan et al., 2019).

### Evaluation: differentiation across treatments or time points

To assess the effectiveness of normalization methods in differentiating across treatments or time points, we calculated effect size (R² value) for each normalized dataset using PERMANOVA (adonis2 function, vegan package in R; Oksanen et al., 2022). This metric reflects the proportion of variance explained by differences in time points or treatments. Effect size was only reported for datasets with statistically significant differentiation across time points or treatments. Additionally, differentiation between treatments was verified using ANOSIM (also from the vegan package in R), serving as a complementary method to confirm observed distinctions in treatment groups.

Significantly altered metabolites in neuronal cells exposed to chlorpyrifos across time points, as identified in both RP positive and RP negative datasets after PQN or Quantile normalization, were uploaded to MetaboAnalyst 6.0 (Pang et al., 2024) to identify associated KEGG pathways. Further details on the data visualization are provided in Supplementary Document 1.

### Evaluation: feature consistency in QC samples

The relative standard deviation (RSD) was calculated for each feature in normalized omics datasets. The number of features with an RSD less than 0.2, i.e., consistent features, was used to demonstrate if normalization methods improved feature consistency in QC samples (Cajka & Fiehn, 2016). To compare across different omics datasets, the number of features with an RSD less than 0.2 for each normalized dataset was compared to its pre-normalized dataset as a metric to quantify improvement following normalization.

### Identification of significant features

The number of significant features was determined sequentially through each feature by building a separate generalized additive model (GAM) with consideration of time and time-treatment interaction as smoothing effects (mgcv package in R) (Wood, 2011). The false discovery rate (FDR) was calculated to correct for multiple comparisons using the p.adjust function, Benjamini & Hochberg adjustment method in R (Benjamini & Hochberg, 1995). FDR less than 0.1 was considered as significant altered features across treatments.

###  Script availability

Detailed R scripts for normalization, evaluation of treatment/time differentiation, QC feature consistency, significant features identification, and plotting are provided in Supplementary Document 1.

## Results

### QC feature consistency and differentiation across timepoints or treatments in normalized metabolomics datasets

**Fig. 2:**
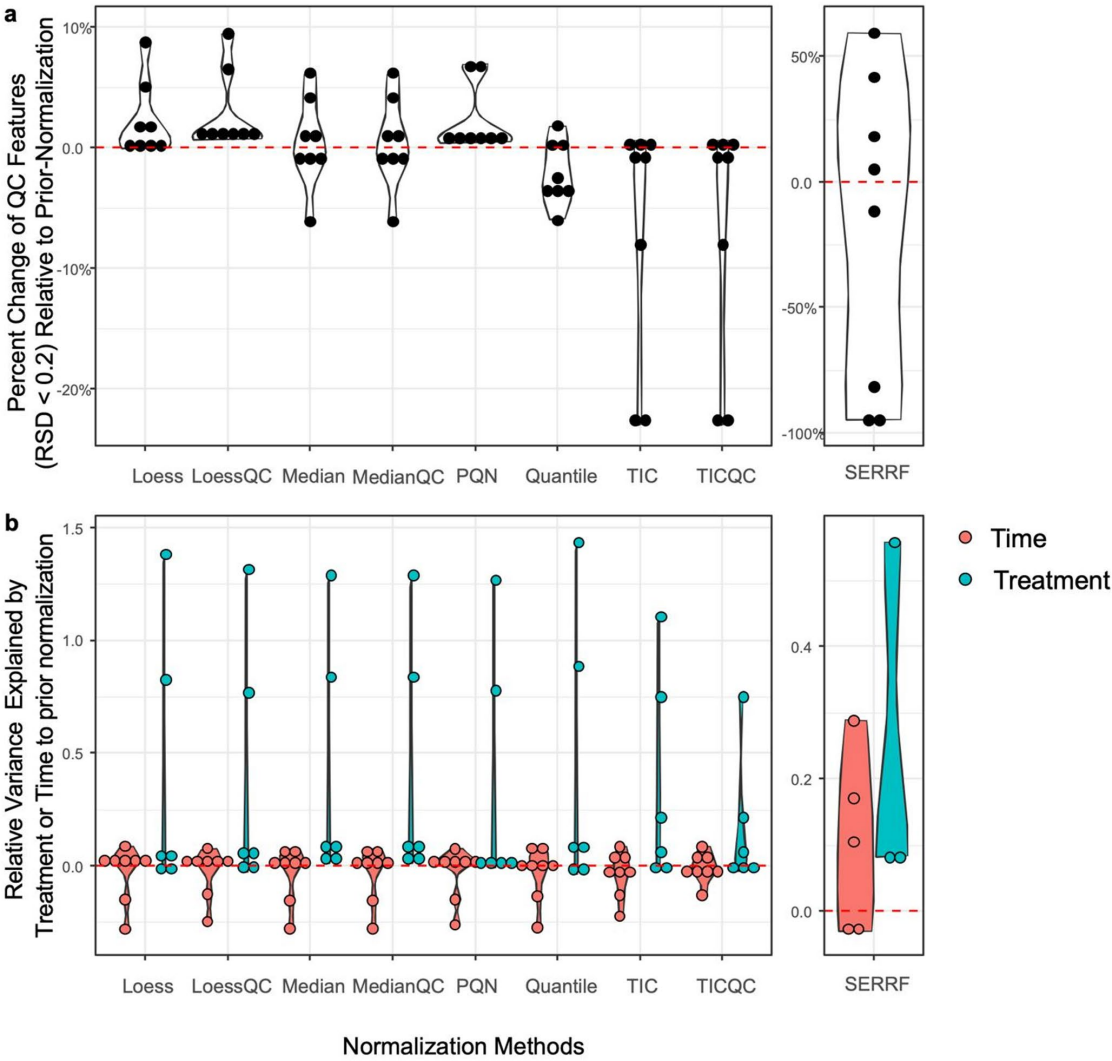
Metabolomics datasets. The effectiveness of each normalization method was evaluated based on two criteria: **a** feature consistency in QC samples, defined as the percent change in consistent QC features (RSD < 0.2) following normalization; and **b** the relative variance explained by treatment or time after normalization. Individual datapoints represent the neuron and cardiomyocyte datasets. Datasets in which variance could not be significantly explained by time or treatment variation (PERMANOVA, *p* ≥ 0.5) were excluded. Note that a different scale was used for the SERRF normalization method to emphasize its distinct performance relative to the other methods

The effectiveness of each normalization method on metabolomics datasets was evaluated based using QC feature consistency and the relative variance explained by treatment or time following normalization (Fig. 2a), assuming the better normalization methods could improve QC feature consistently across datasets. Normalized datasets from four primary human cardiomyocyte and four primary human neuronal samples were included as datapoints in the comparison. In cardiomyocytes and neurons, comparison of metabolomics datasets before normalization showed that QC feature was more consistent in neurons (median QC RSD ≈ 0.19) than in cardiomyocytes (median QC RSD ≈ 0.35) (Table S1). Following normalization, Loess, LoessQC, and PQN improved QC feature consistency, as indicated by reducing median RSD values in QC samples and an increase in the number of features with RSD < 0.2 relative to pre-normalization levels across datasets, supporting their effectiveness in reducing systematic variability (Table 1).

**Table 1:** Summary comparison of normalization methods performance across metabolomics, lipidomics, and proteomics datasets

Norm. Method	# Sig-nificant by Treat.	Median RSD	Relative Δ # RSD < 0.2	Avg. ΔR^2^/R^2^ (Pre-Norm) by Treat.	Avg. ΔR^2^/R^2^ (Pre-Norm) by Time
Metabolomics					
Pre-normalization	6/8	0.277 (0.038)	0 (0)	0 (0)	0 (0)
SERRF	3/8	0.207 (0.084)	− 0.198 (0.221)	0.24 (0.159)	0.102 (0.06)
LoessQC	6/8	0.272 (0.037)	0.027 (0.012)	0.366 (0.226)	− 0.024 (0.038)
Loess	6/8	0.273 (0.037)	0.022 (0.011)	0.38 (0.241)	− 0.025 (0.044)
PQN	6/8	0.273 (0.038)	0.022 (0.01)	0.355 (0.221)	− 0.028 (0.04)
Median	6/8	0.275 (0.037)	0.004 (0.013)	0.395 (0.22)	− 0.029 (0.043)
MedianQC	6/8	0.275 (0.037)	0.004 (0.013)	0.395 (0.22)	− 0.029 (0.043)
Quantile	6/8	0.276 (0.036)	− 0.021 (0.009)	0.411 (0.249)	− 0.028 (0.042)
TICQC	6/8	0.282 (0.039)	− 0.067 (0.036)	0.169 (0.122)	− 0.006 (0.023)
TIC	6/8	0.282 (0.04)	− 0.068 (0.036)	0.354 (0.191)	− 0.034 (0.035)
Lipidomics					
Pre-normalization	3/4	0.15 (0.019)	0 (0)	0 (0)	0 (0)
LoessQC	3/4	0.14 (0.024)	0.044 (0.003)	0.083 (0.107)	− 0.139 (0.013)
PQN	3/4	0.141 (0.021)	0.04 (0.007)	0.123 (0.032)	− 0.147 (0.027)
Loess	2/4	0.142 (0.021)	0.027 (0.016)	0.121 (0.16)	− 0.16 (0.063)
Median	2/4	0.146 (0.019)	0.02 (0.005)	0.045 (0.115)	− 0.102 (0.054)
MedianQC	2/4	0.146 (0.019)	0.02 (0.005)	0.045 (0.115)	− 0.102 (0.054)
TIC	3/4	0.155 (0.033)	− 0.062 (0.104)	0.175 (0.074)	− 0.115 (0.03)
TICQC	3/4	0.155 (0.033)	− 0.062 (0.104)	0.175 (0.074)	− 0.115 (0.03)
Quantile	2/4	0.162 (0.022)	− 0.096 (0.032)	0.076 (0.092)	− 0.138 (0.014)

	Treatment Effect (p)	ΔR²/R² (Pre-Norm) by Treat.	ΔR²/R² (Pre-Norm) by Time
Proteomics			
Cardiomyocyte			
Prior Normalization	0.52	0	0
Median	0.013	0.712	− 0.008
PQN	0.014	0.701	− 0.011
Loess	0.01	0.671	− 0.015
VSN	0.016	0.713	− 0.016
Quantile	0.013	0.708	− 0.02
TIC	0.031	0.698	− 0.029
Neuron			
Prior Normalization	0.027	0	0
Loess	0.039	− 0.03	0.077
PQN	0.04	− 0.05	0.058
Median	0.043	− 0.058	0.066
TIC	0.041	− 0.067	0.021
Quantile	0.045	− 0.069	0.06
VSN	0.055	− 0.081	0.126

The table summarizes the number of datasets with significant treatment effects (# significant by Treat.), the average of median QC feature relative standard deviations (Median RSD) across datasets, and the average relative change in variance explained by treatment or time following normalization (Avg. ΔR²/R² (Pre-Norm) by Treat., Avg. ΔR^2^/R^2^ (Pre-Norm) by Time) in metabolomics and lipidomics. All comparisons except for proteomics were performed on combined datasets from neurons and cardiomyocytes. For proteomics, we assessed the statistical significance of the treatment effect (Treatment Effect (*p*)) and the change in variance explained by treatment or time following normalization (ΔR²/R² (Pre-Norm) by Treat., ΔR^2^/R^2^ (Pre-Norm) by Time) separately for neuronal and cardiomyocyte datasets

Time accounted for over 20% of variance, while treatment typically explained less than 5% variance in feature space (Table S1). The relative variance explained by time or treatment in each dataset following normalization, compared to pre-normalization, is shown as individual data points (Fig. 2b). Datasets in which variance was not significantly explained by time or treatment (*p* < 0.05) were excluded from the comparison. In most metabolomic datasets, normalization increased the variance explained by either time or treatment. However, in two datasets, normalization substantially increased the variance explained by treatment, while concurrently reducing the variance explained by time (Fig. 2b).

However, SERRF normalization failed to improve the number of consistent QC features in 50% of datasets (Fig. 2a). In addition, variance was not significantly explained by treatment in 62.5%, indicating that SERRF is a more dataset-dependent normalization method for these metabolomics datasets. Nevertheless, when effective, SERRF normalization increased consistent QC features by up to 50%, compared to a maximum of 10% improvement observed with other normalization methods.

### QC feature consistency and differentiation across timepoints or treatments in normalized lipidomics datasets

**Fig. 3:**
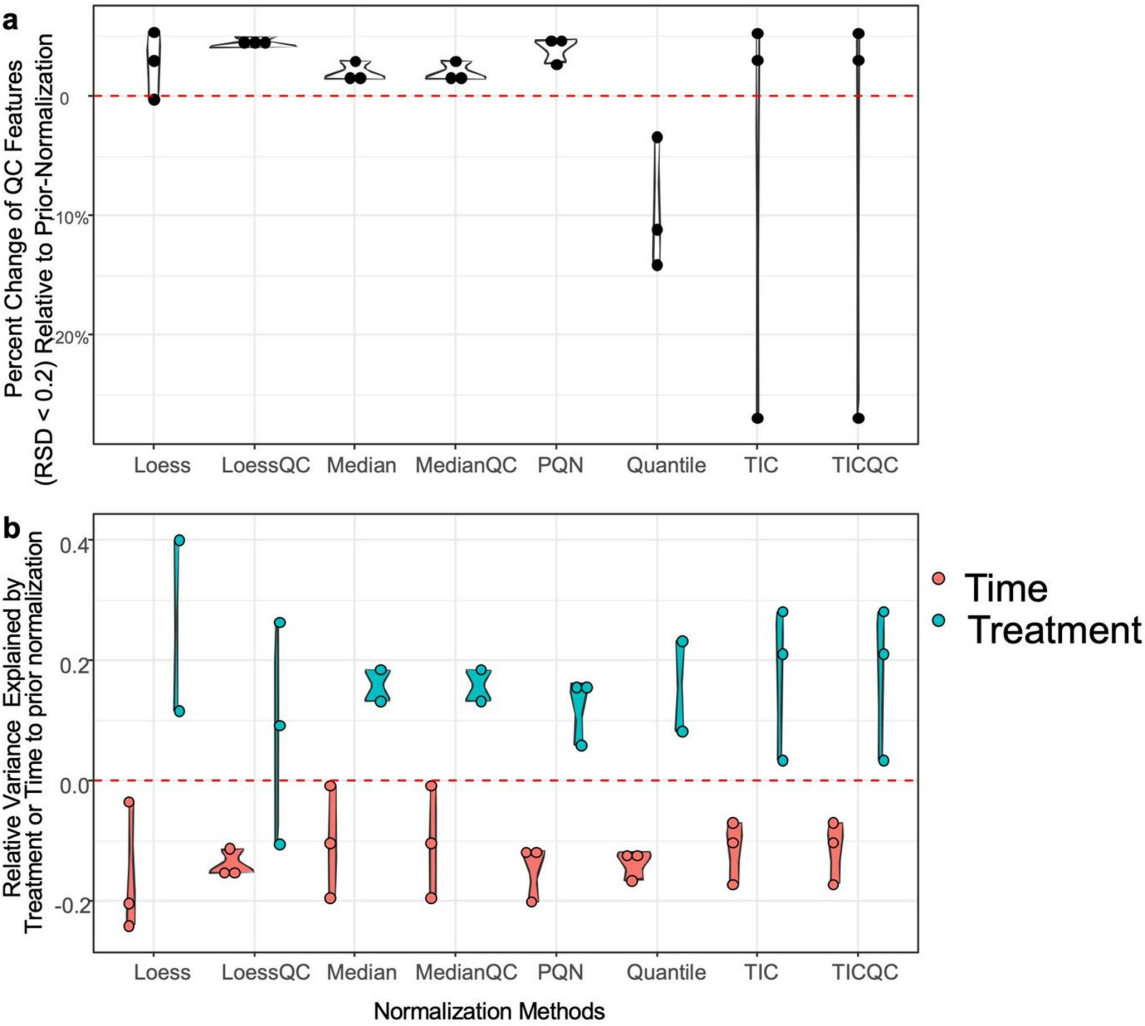
Lipidomics datasets. The effectiveness of each normalization method was evaluated using two criteria: **a** feature consistency in QC samples, defined as the percent change in consistent QC features (RSD < 0.2) following normalization; and **b** the relative variance explained by treatment or time following normalization. Individual datapoints represent the neuron and cardiomyocyte datasets. Datasets in which variance was not explained by time or treatment (PERMANOVA, *p* ≥ 0.5) were excluded

Lipidomics datasets, measured in both positive and negative modes from the same cell lysate as metabolomics, showed improved QC feature consistency following normalization with Loess, LoessQC, Median, MedianQC, and PQN (Fig. 3a; Table 1). Among these five normalization methods, PQN and LoessQC yielded the lowest median RSD values (Table 1). Cardiomyocytes exhibited slightly lower QC feature variability (median QC RSD ≈ 0.11) compared to neurons (median QC RSD ≈ 0.17) (Table S2).

In these lipidomics datasets, time accounted for over 20% of the variance, while treatment typically explained less than 5% (Table S2). Changes in variance was visualized as individual data points, excluding datasets where time or treatment effects were not significant (*p* < 0.05) (Fig. 3b). Overall, normalization reduced the variance explained by time while increasing the variance explained by treatment (Fig. 3b; Table 1).

### Differentiation across timepoints or treatments in normalized proteomics datasets

QC feature consistency was not assessed in the proteomics datasets, as pooled QC samples were not included in the experimental design. Instead, we evaluated the effect of normalization on datasets from the two cell types. Comparing feature intensity, cardiomyocytes showed much higher feature intensity variability than neuron datasets (Fig. S1).

**Fig. 4:**
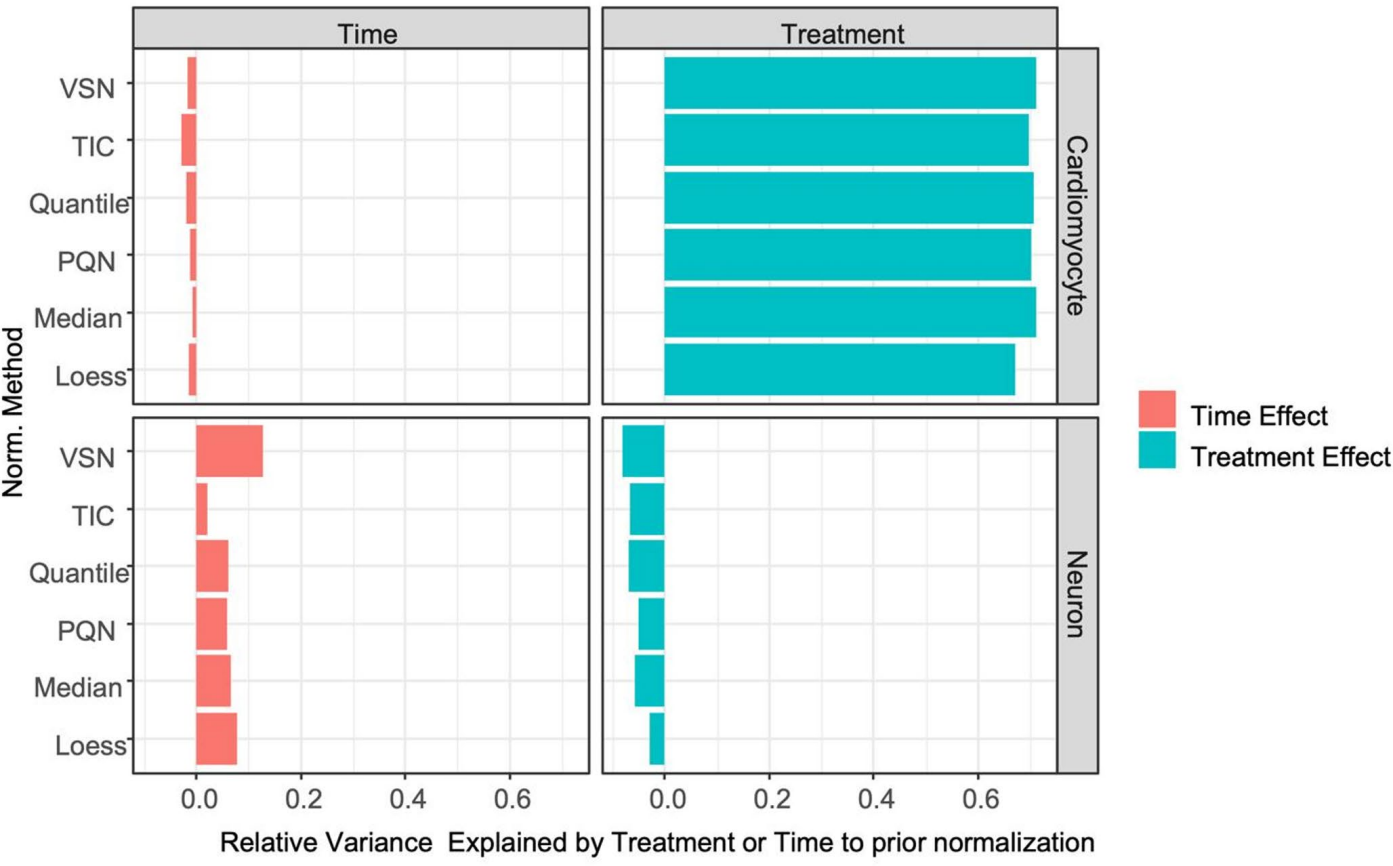
The effectiveness of each normalization method in proteomics datasets, acquired from cardiomyocyte and neuron samples, was evaluated based on the relative variance explained by treatment or time following normalization. Individual bars represented the neuron and cardiomyocyte datasets, variance in time and treatment effects after normalization is shown in orange and blue, respectively

Time initially accounted for a substantially larger portion of variance (≈ 17% R²) compared to treatment (≈ 3% R²) (Table S3). In cardiomyocyte datasets, normalization reduced the variance explained by time while greatly increasing the variance explained by treatment (Fig. 4). Conversely, in neuron datasets, normalization increased the variance explained by time while reducing that explained by treatment. Among the normalization methods, PQN, Median, and Loess were the least likely to reduce variance explained by time in cardiomyocyte datasets or by treatment in neuron datasets (Fig. 4; Table 1).

### Downstream effects of normalization methods: significant feature size and related pathways

**Fig. 5:**
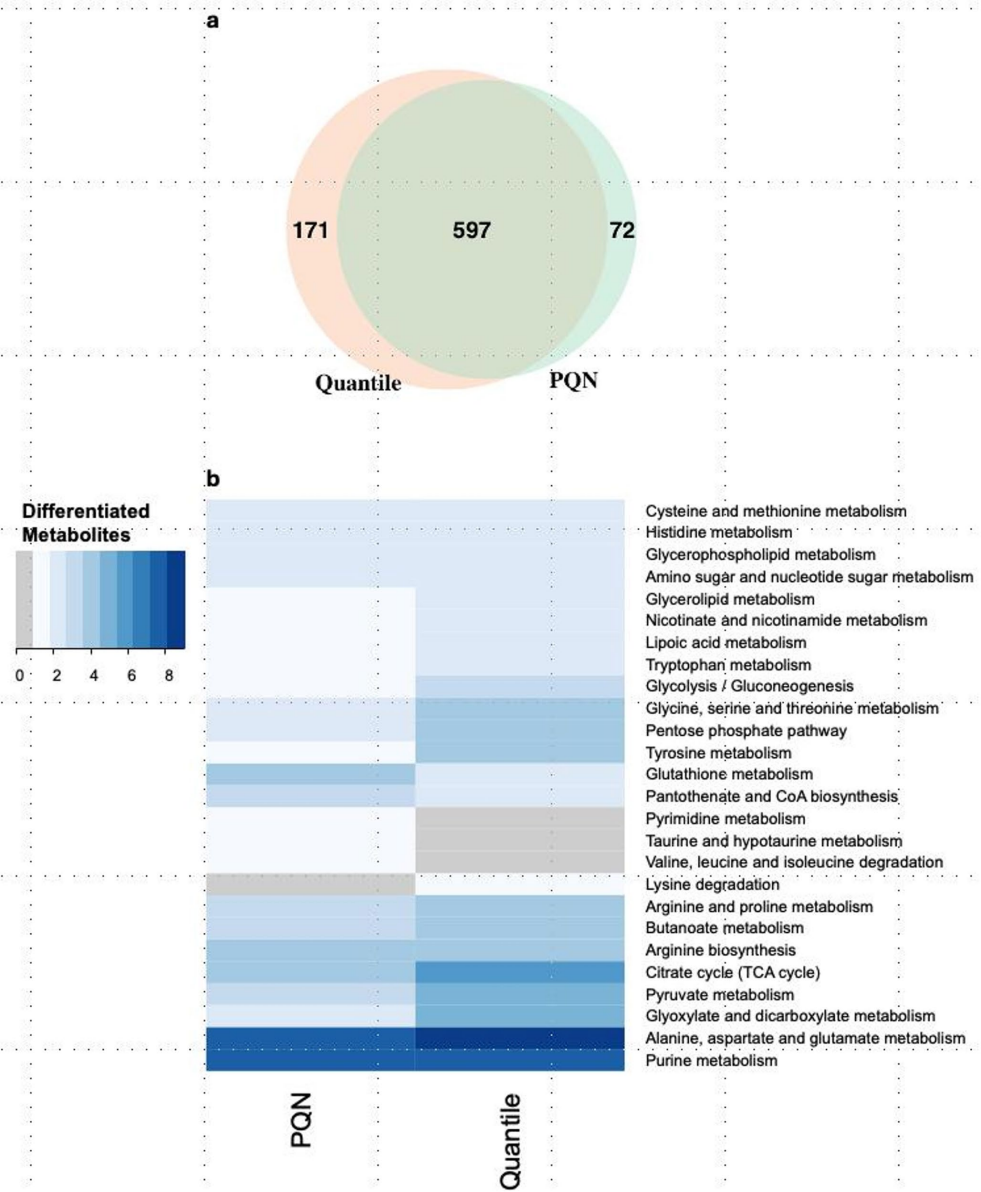
We compared significantly altered metabolites identified from PQN-normalized and Quantile-normalized reverse-phase (RP) positive and RP negative datasets in neuron cells exposed to chlorpyrifos. PQN normalization represented the better-performing method, while Quantile normalization was one of the least effective. **a** Venn diagram showing that Quantile normalization identified 15% more significant metabolites than PQN normalization across all time points. **b** Heatmap comparing the KEGG pathways associated with the altered metabolites, colored by the number of annotated metabolites in each pathway. Although both methods identified largely overlapping KEGG pathways, PQN uniquely detected three amino acid metabolism pathways not identified in the Quantile-normalized datasets

The number of significant features between treatments and their associated functions depends on the chosen normalization method. To illustrate this, we compared two reverse-phase metabolomic neuron datasets to examine how significantly differentiated features (*FDR* < 0.1), and their associated pathways varied between PQN normalization—which consistently improved QC feature consistency—and Quantile normalization, which was least effective. Quantile normalization identified 15% more significant metabolites than PQN normalization across all time points (Fig. 5a). Although the pathways associated with these altered metabolites were largely consistent, PQN uniquely enabled the detection of three KEGG pathways related to amino acid metabolism that were not observed in the Quantile-normalized datasets (Fig. 5b).

## Discussion

### Observing normalization effects across datasets

Normalization enhances feature consistency in QC samples, reduces analytical variability, and facilitates the identification of biologically significant features. Common metrics for evaluating normalization effectiveness include PCA dispersion, median RSD in QC samples, and the total number of consistent features (Kirwan et al., 2013; Wang et al., 2013). In this study, we used the number of consistent features following normalization as a key indicator of reduced systematic error, and observed changes in the variance explained by time or treatment across datasets following normalization. The multi-omics datasets originated from the same samples and lysates (Breidenbach et al., 2024), enabling a fair evaluation of normalization approaches by minimizing inter-sample variability.

Chlorpyrifos (an organophosphate pesticide) and carbaryl (an N-methyl carbamate pesticide), both known acetylcholine-active compounds (Eaton et al., 2008; Gunasekara et al., 2008), induced strong biological effects at tested exposure levels (Rivera et al., 2024). Among the two cell types studied, cardiomyocyte datasets exhibited greater variability, especially in metabolomics and proteomics, suggesting higher noise levels, and the noisy mass spectra could mask peaks of biological importance (Du et al., 2008). To ensure transparency and robustness, normalization methods were evaluated using predefined metrics with the assumption that the benchmark outcome varies in terms of data characteristics (Brombacher et al., 2025). Given the limited sample size, we did not perform cross-validation, which can bias results toward methods that emphasize increasing variance, i.e., over-fitting the data (Xi et al., 2014). For omics data, meaningful cross-validation for classifying treatment effects typically requires a substantially larger number of biological replicates (Rodríguez-Pérez et al., 2018; Zhang et al., 2019). However, because we examined variance explained by time, treatment, and their interaction, we effectively had only three replicates per condition. Instead of performing cross-validation, we prioritized methods that enhance QC feature consistency and observed how different normalization affected variance explained by time or treatment.

Most normalization methods assume central tendency, constant distribution, and similar systematic biases across feature intensities (Callister et al., 2006; Veselkov et al., 2011). Key differences among normalization methods include whether they alter feature rankings (e.g., VSN), assume a normal distribution or use a reference distribution (Quantile vs. PQN), normalize based on equal total intensity (TIC), and whether they rely solely on QC samples or incorporate all samples. We included widely used normalization methods based on distinct underlying assumptions, but excluded others that shared similar assumptions.

However, these assumptions may not hold because of the complex nature of time-course datasets, which can introduce diverse and unpredictable variation (De Livera et al., 2012; Yang et al., 2020). While few studies evaluated the effectiveness of normalization methods by assessing their classification performance, none have included variance explained by time (Yang et al., 2020). Time effects account for the majority of variance in longitudinal datasets and interact with treatment effects (Ren et al., 2015). Therefore, preserving time-related variance is a key metric for assessing whether normalization introduces additional variation.

In addition to time-course challenges, normalization effects on QC feature consistency varied across omic layers. In metabolomics and lipidomics, PQN and LoessQC yielded consistent improvement in QC feature consistency across datasets while quantile, TIC, and TICQC performed worse in QC consistency. The current proteomics datasets did not include QC samples; QC feature consistency was not evaluated. Although SERRF normalization is gaining popularity for bridging batch effects (Bergmann et al., 2024; Fan et al., 2019; Huang et al., 2021), we found that SERRF substantially improve QC consistency in some metabolomics datasets but was ineffective in others. In several cases, treatment-related variance missed by SERRF was captured by other methods, suggesting that SERRF may obscure genuine biological signals.

SERRF has been shown to be unsuitable for smaller datasets, e.g., < 500 samples, as it has shown signs of overfitting and varied performance (Fan et al., 2019; Zhang et al., 2023).

In metabolomics, normalization generally increased the variance explained by both time and treatment, whereas in lipidomics, it tended to decrease the variance explained by time while concurrently increasing the variance explained by treatment. In proteomics, the effect of normalization was mixed, either increasing or decreasing the variance explained by time depending on the cell types. The decrease of variance explained by time or treatment could suggest a biased normalization result, or over-normalization, which may related to median feature intensity and sample-dependent detection limits (Brombacher et al., 2025). The introduction of biases could obscure meaningful biological variation, leading to misleading conclusions (Wehrli et al., 2019)

### Downstream effects following normalization

We identified PQN and LoessQC as the most robust normalization methods across all omic layers while Quantile normalization was often the least effective in terms of QC feature consistency. When comparing PQN and Quantile, we observed that both these methods identified similar KEGG pathways, suggesting that the choice of normalization method did not substantially alter overall pathway annotations, consistent with a recent study (Carvalho et al., 2024). However, despite this similarity, PQN normalization uncovered three dysregulated biochemical pathways related to amino acid degradation that Quantile normalization did not. This indicates that PQN is overall more effective than Quantile normalization in capturing biologically relevant responses.

## Conclusion

Our study highlights the importance of selecting appropriate normalization methods across omics datasets before multi-omics integration. By systematically evaluating commonly used methods using multi-omics datasets derived from a single experiment—including those from the same cell lysate, we identified several key assessment criteria. These criteria emphasize maximizing QC feature consistency and preserving critical data structures as variance explained by time or treatment.

Normalization methods can have varying impacts on multi-omics datasets, influenced by factors such as cell type, signal strength, and inherent data variation. Considering all these factors, we identified that PQN and LoessQC normalization in metabolomics and lipidomics, and PQN, Median, and Loess normalization in proteomics were the top normalization methods. In each case, these methods consistently improved QC feature consistency without compromising underlying data structure. Selecting appropriate normalization methods enhances the reliability of multi-omics data integration and supports more accurate biological interpretations. We recommend a thorough evaluation of normalization effects on individual datasets prior to multi-omics integration in a time-course study.

## Supplementary Information

Below is the link to the electronic supplementary material.

Supplementary material 1 (TIFF 9426.6 kb)

Supplementary material 2 (PDF 187.1 kb)

Supplementary material 3 (PDF 1144.4 kb)

Supplementary material 4 (PDF 229.4 kb)

Supplementary material 5 (CSV 7.8 kb)

Supplementary material 6 (CSV 2.8 kb)

Supplementary material 7 (CSV 1.3 kb)

Supplementary material 8 (CSV 3.3 kb)

## Data Availability

Data that support the findings of this study have been deposited in the MassIVE database with the accession code MSV000097689 (Lipidomics), MSV000097682 (Proteomics), and MSV000097702 (Metabolomics). Normalization and evaluation R script are described in the supplementary document 1 and provided in the supplementary files.
