# Supplementary figures and images for: Evaluation of normalization strategies for mass spectrometry-based multi-omics datasets
Source: Metabolomics. 2025 Jul 1;21(4):98. doi: 10.1007/s11306-025-02297-1 (PMC12214035; doi:10.1007/s11306-025-02297-1)

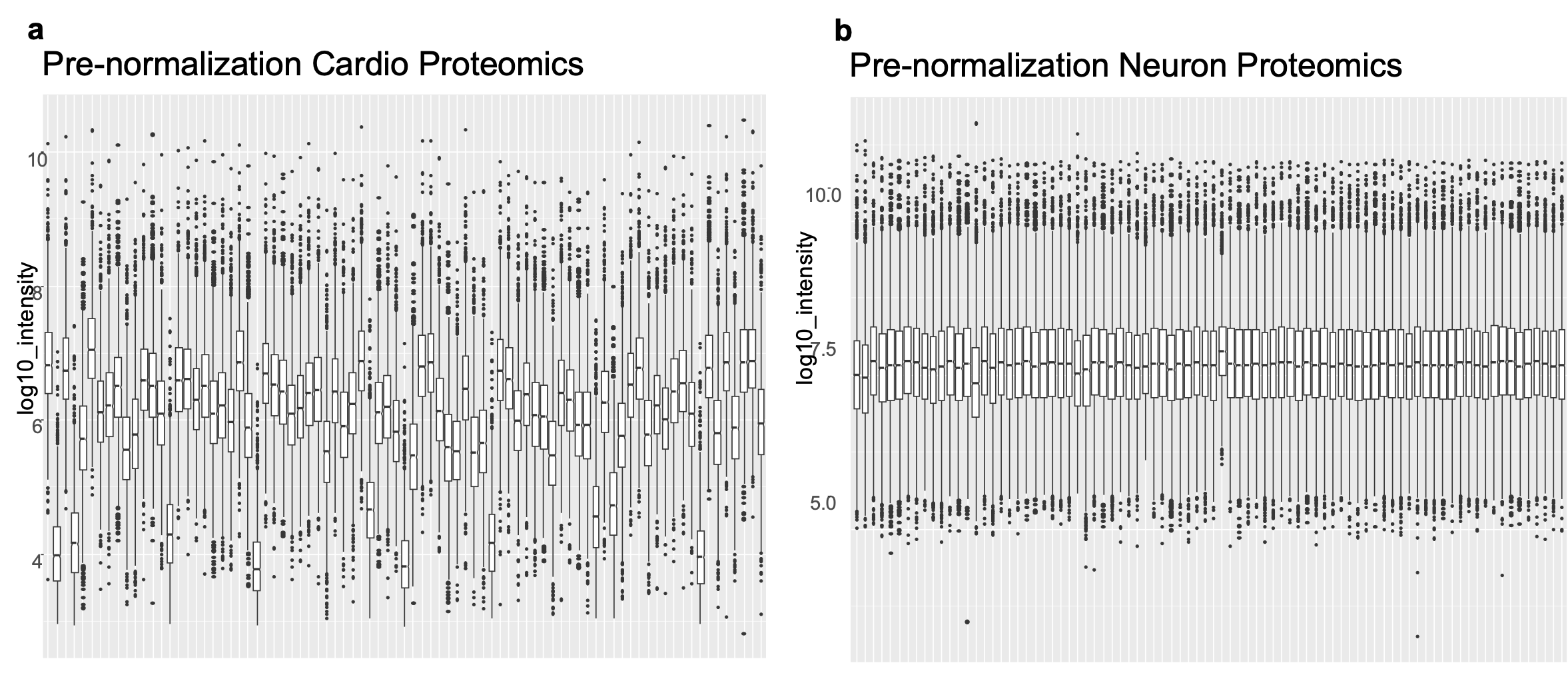

Supplement: Supplementary file 1 — Supplementary material 1 (TIFF 9426.6 kb) [file 11306_2025_2297_MOESM1_ESM.tiff]
