# Supplementary material for: Evaluation of normalization strategies for mass spectrometry-based multi-omics datasets
Source: Metabolomics. 2025 Jul 1;21(4):98. doi: 10.1007/s11306-025-02297-1 (PMC12214035; doi:10.1007/s11306-025-02297-1)
Supplement: Supplementary file 2 — Supplementary material 2 (PDF 187.1 kb) [file 11306_2025_2297_MOESM2_ESM.pdf]

Supplementary Document 1

2. Method

2.1 Cell lysis, sample extraction, data collection for metabolomics, lipidomics, and proteomics

For cell lysis, frozen adherent cells were reconstituted in a monophasic solvent (60% n-butanol, 20% acetonitrile, 20% water). The bead-bound proteins were separated magnetically, and the supernatant was aliquoted for lipidomic and metabolomic analyses. Proteomic data were acquired on a Dionex 3000 UHPLC coupled to a tribrid Eclipse mass spectrometer using reversed-phase chromatography on a C18 EasySpray column with a 65-min gradient and data dependent acquisition (DDA) performed. Lipidomic and metabolomic analyses were performed on a Dionex 3000 UHPLC interfaced with an Exploris 480 MS using reversed-phase chromatography (C8 and Cortecs T3 C18 columns, respectively) under optimized gradients.

2.2 Data preprocessing

In the metabolomics datasets, features were filtered based on the following criteria: relative standard deviation (RSD) < 0.3, presence of MS/MS data, Delta Mass < 5 m/z, and a signal intensity at least 3 standard deviations above the background. For lipidomics, features were filtered to ensure they were 3 standard deviations above background in at least 80% of samples, with additional half-minimum gap filling for data imputation. In the proteomics datasets, features with missing values in more than one-third of samples across any time point or treatment group were removed. Missing data in proteomics were then imputed using the Classification and Regression Trees (CART) method from the R mice package (Buuren and Groothuis-Oudshoorn 2011), which facilitates gap filling by accounting for non-linear relationships and complex interactions in the data while minimizing imputation for sparsely populated features.

2.3 Data visualization

For visual comparison of different normalization methods, a heatmap was generated using heatmap.2 from the gplots package in R. Volcano plots were created to display log2 fold changes across each time point, with corresponding FDR values for each feature, highlighting significant alterations. All the other visualizations, including quadrant plots, bar chart, and volcano plots, were generated using the ggplot2 package in R.

2.4 Evaluation: Dispersion Between Biological Replicates

Average dispersion between biological replicates was calculated using the betadisper function from the vegan package in R. This function measured multivariate homogeneity of group dispersions (variances) as the average distance of group members from the group centroid within each time and treatment group. To compare normalization methods, relative group dispersions were standardized against the media control group and evaluated using a linear mixed effect model (lmer) from the lme4 Package in R (Bates et al. 2015). Detail dispersion

calculation and evaluation for each layer of omics are shown in *Supplementary_diffusion_evaluation.pdf.*

3. Result

3.1 Normalization methods affect the dispersion between replicates

For metabolomics, current normalization methods did not increase dispersion between replicates. However, SERRF significantly reduced dispersion in both biological replicates and QC samples in the more variable cardiomyocyte datasets ($p < 0.001$), an effect not observed in neuron datasets. In lipidomics datasets, most normalization methods slightly increased dispersion among biological replicates, with a significant increase observed for Loess ($p < 0.001$) and PQN ($p < 0.05$). Notably, none of the normalization methods increased dispersion in lipidomics QC samples. In proteomics datasets, dispersion among biological replicates remained unaffected by normalization methods (Table S4). Detail dispersion evaluation and visualization for each layer of omics are shown in *Supplementary_diffusion_evaluation.pdf*.

4. Discussion

4.1 Normalization methods affect the dispersion between replicates

Assessing dispersion between QC and biological replicates is crucial to ensure that normalization methods do not introduce additional variability. Our results showed that most normalization methods did not increase dispersion in metabolomics and proteomics datasets. However, in lipidomics datasets, normalization sometimes increased variation among biological replicates which have inherently higher feature variation. Despite the sporadic increase in variation among biological replicates, the consistent improvement in QC feature consistency underscored the benefit of normalization. However, SERRF normalization significantly reduced dispersion between replicates in cardiomyocyte metabolomics datasets. This reduction in variability suggested that SERRF is effective at minimizing technical variation in datasets with higher inherent variation (Fan et al. 2019). However, SERRF did not reduce dispersion between replicates in neuron datasets which had inherently lower variability and stronger signals. This disparity indicated that the performance of SERRF normalization depends on the specific characteristics of the dataset.

5. Supplementary Script

Supplementary scripts are available in *Supplementrary_script.pdf*, including the scripts for normalization and evaluating normalization effectiveness.

Figure S1. Proteomics feature intensity distribution measured in (a) cardiomyocyte and (b) neuron cell lines.

Table S1. Metrics for evaluating the effect of normalization in metabolomics datasets, including the total number of significant differentiated features across all time points ($p.adj < 0.1$), significantly differentiation between treatment groups (PERMANOVA: Adonis2_P_trt) and between time groups (PERMANOVA: Adonis2_P_time). And the variance explained by treatment (R2_trt), the variance explained by time (R2_time), and the variance explained by time-treatment interaction (R2_time_trt). The separation between time group was further confirmed using ANOSIM (Anosim_P_time). QC feature consistency measured by median Relative standard deviation (RSD) (rsd_median) and the number of features with RSD < 0.2 (rsd_0.2) were also included.

Table S2. Metrics for evaluating the effect of normalization in lipidomics datasets, including the total number of significant differentiated features across all time points ($p.adj < 0.1$), significantly differentiation between treatment groups (PERMANOVA: Adonis2_P_trt) and between time groups (PERMANOVA: Adonis2_P_time). And the variance explained by treatment (R2_trt), the variance explained by time (R2_time), and the variance explained by time-treatment interaction (R2_time_trt). The separation between time group was further confirmed using ANOSIM (Anosim_P_time). QC feature consistency measured by median Relative standard deviation (RSD) (rsd_median) and the number of features with RSD < 0.2 (rsd_0.2) were also included.

Table S3. Metrics for evaluating the effect of normalization in proteomics datasets, including the total number of significant differentiated features across all time points ($p.adj < 0.1$), significantly differentiation between treatment groups (PERMANOVA: Adonis2_P_trt) and between time groups (PERMANOVA: Adonis2_P_time). And the variance explained by treatment (R2_trt), the variance explained by time (R2_time), and the variance explained by time-treatment interaction (R2_time_trt). The separation between time group was further confirmed using ANOSIM (Anosim_P_time).

Table S4. Dispersion between replicates at each timepoints and treatment group was estimated relative to the media control dispersion. The relative dispersions (Estimate) and significance (Pr(>|t|)) were compared with the dispersion before normalization and presented for each normalization methods.

Reference

Bates, D., Mächler, M., Bolker, B., & Walker, S. (2015). Fitting Linear Mixed-Effects Models Using lme4. *Journal of Statistical Software*, *67*, 1–48. https://doi.org/10.18637/jss.v067.i01

Buuren, S. van, & Groothuis-Oudshoorn, K. (2011). mice: Multivariate Imputation by Chained Equations in R. *Journal of Statistical Software*, *45*, 1–67. https://doi.org/10.18637/jss.v045.i03

Fan, S., Kind, T., Cajka, T., Hazen, S. L., Tang, W. H. W., Kaddurah-Daouk, R., et al. (2019). Systematic Error Removal Using Random Forest for Normalizing Large-Scale Untargeted Lipidomics Data. *Analytical Chemistry*, *91*(5), 3590–3596. https://doi.org/10.1021/acs.analchem.8b05592
