# Supplementary material for: Evaluation of normalization strategies for mass spectrometry-based multi-omics datasets
Source: Metabolomics. 2025 Jul 1;21(4):98. doi: 10.1007/s11306-025-02297-1 (PMC12214035; doi:10.1007/s11306-025-02297-1)
Supplement: Supplementary file 3 — Supplementary material 3 (PDF 1144.4 kb) [file 11306_2025_2297_MOESM3_ESM.pdf]

# Norm_MS_Dispersion_Evaluation

Chi Yen Tseng

March 24, 2025

```
setwd("~/Documents/Projects/Inception/Norm_manuscript/")
```

Load packages

```
library(tidyverse)
library(gridExtra)
library(grid)
library(svglite)
library(gt)
library(lmerTest)
```

## Metabolomics

### Cardio

Read Cardio:Metabolomics evaluation metrics

```
# Metabolomics
# for combine all Norm evaluation result
Eval_norm_method_RPpos <- readRDS("/Users/chiyen_tseng/Documents/Projects/Inception/metabolomics/Cardio
# RPpos (there is one QC sample drift from the baseline which might affect SERRF normalization)
Eval_norm_method_RPneg <- readRDS("/Users/chiyen_tseng/Documents/Projects/Inception/metabolomics/Cardio
# RPneg (batch effect, group left is going along with almost all the QC samples; only use group left ab
Eval_norm_method_Hilicpos <- readRDS("/Users/chiyen_tseng/Documents/Projects/Inception/metabolomics/Card
# HILIC pos has haf of re-injection
Eval_norm_method_Hilicneg <- readRDS("/Users/chiyen_tseng/Documents/Projects/Inception/metabolomics/Card
# quantile GAM model failed becasue quanitle norm is very unstable
```

Combine cardio metabolomics dispersion

```
# combine cardio metabiolomcis dispersion
RPpos.dis <- Eval_norm_method_RPpos$dispersion
RPneg.dis <- Eval_norm_method_RPneg$dispersion
HILICpos.dis <- Eval_norm_method_Hilicpos$dispersion
HILICneg.dis <- Eval_norm_method_Hilicneg$dispersion

Cardio_metabolomics_dis <- list(RPpos.dis, RPneg.dis, HILICpos.dis, HILICneg.dis)
Cardio_metabolomics_dis <- do.call("rbind",Cardio_metabolomics_dis)
```

Making linear mixed effect model with dataset, time, and Trt as random effect, and normalization method as fixed effect

```
# if dispersion changes between normalization methods
# check dispersion is dependent on norm method

dispersion_compare_data <- as.data.frame(Cardio_metabolomics_dis)
dispersion_compare_data$dataset <- as.factor(dispersion_compare_data$dataset)
dispersion_compare_data <- dispersion_compare_data %>% filter(Trt != "PooledQC") %>% filter(!dataset %i

# make linear mix effect model
p5 <- lmer(relative_dispersion ~ norm_method + (1 | dataset:Time) + (1 | dataset:norm_method) + (1 | da
           data = dispersion_compare_data)

plot(p5)
```

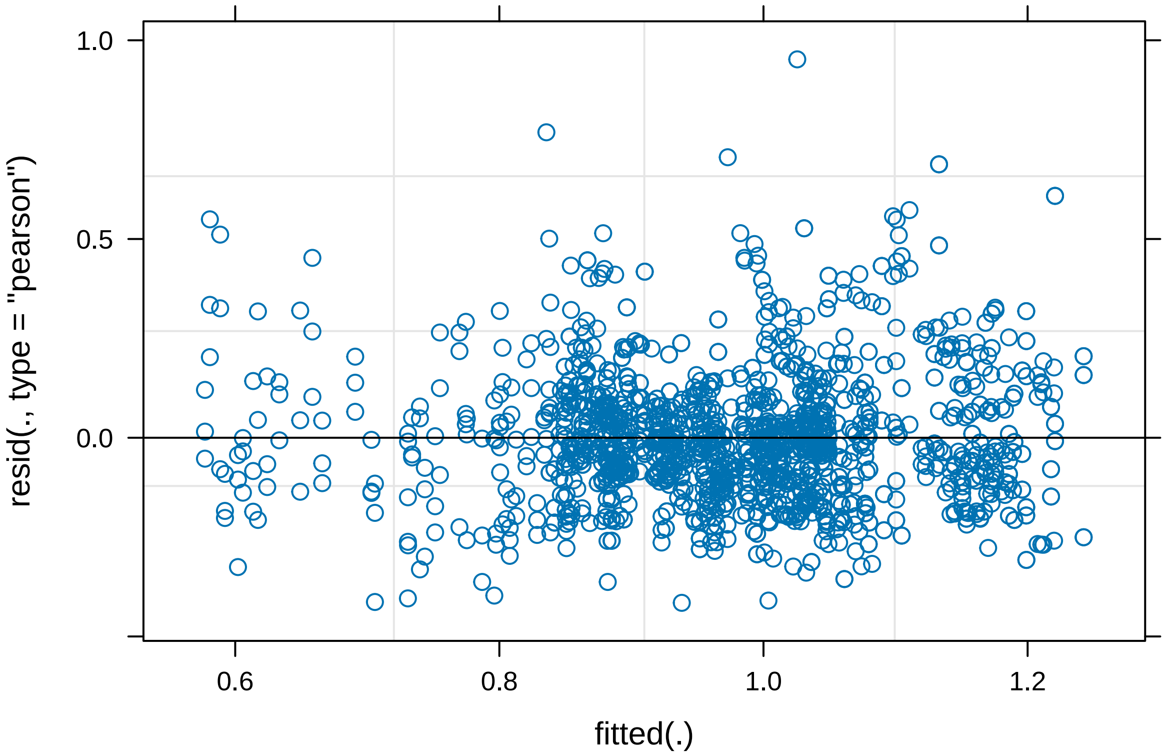


```
qqnorm(resid(p5))
```

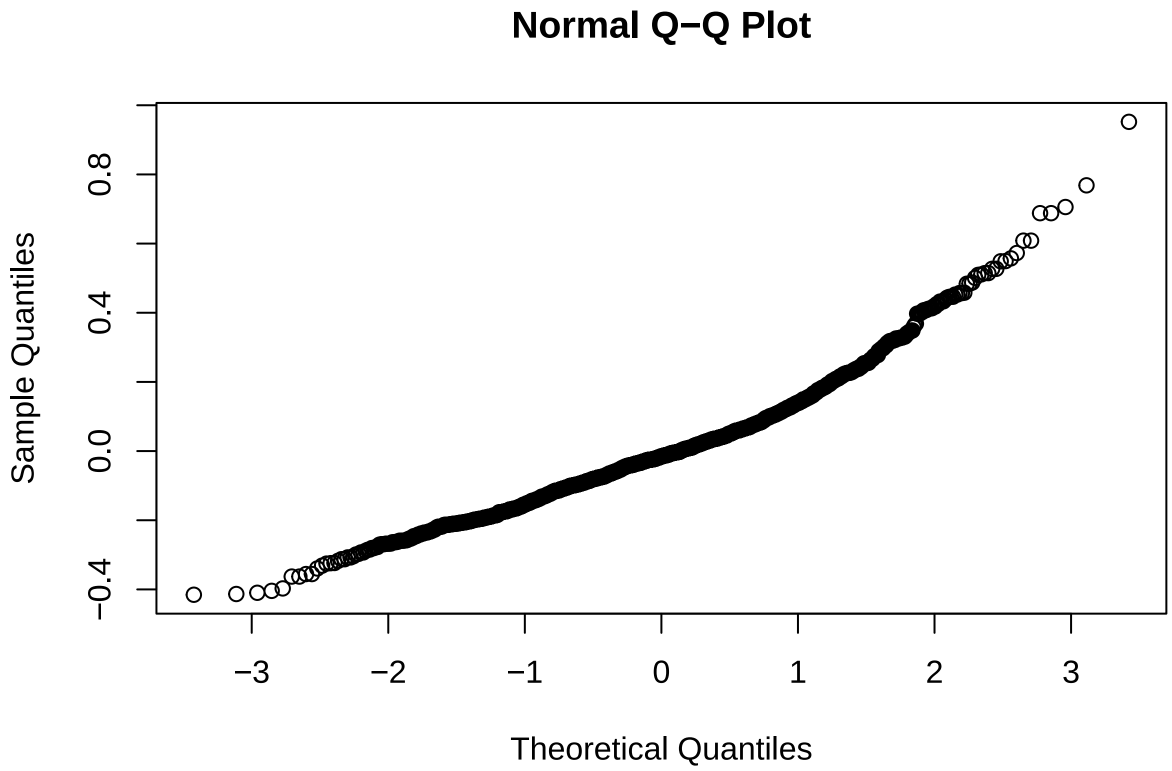


```r
summary(p5)
```

```
## Linear mixed model fit by REML. t-tests use Satterthwaite's method [
## lmerModLmerTest]
## Formula: relative_dispersion ~ norm_method + (1 | dataset:Time) + (1 |
##     dataset:norm_method) + (1 | dataset:Trt)
##    Data: dispersion_compare_data
##
## REML criterion at convergence: -1107.7
##
## Scaled residuals:
##     Min      1Q  Median      3Q     Max
## -2.5235 -0.6214 -0.0929  0.4626  5.7832
##
## Random effects:
##  Groups              Name        Variance  Std.Dev.
##  dataset:norm_method (Intercept) 0.0009079 0.03013
##  dataset:Time        (Intercept) 0.0069668 0.08347
##  dataset:Trt         (Intercept) 0.0040380 0.06355
##  Residual                        0.0270979 0.16461
## Number of obs: 1620, groups:
## dataset:norm_method, 20; dataset:Time, 18; dataset:Trt, 6
##
## Fixed effects:
##                      Estimate Std. Error       df t value Pr(>|t|)
```

```
## (Intercept)          0.97080    0.04100 17.81983  23.676 6.47e-15 ***
## norm_methodloess     0.03169    0.03525  9.01079   0.899 0.391985
## norm_methodloessQC   0.02026    0.03525  9.01079   0.575 0.579502
## norm_methodmedian    0.06032    0.03525  9.01079   1.711 0.121169
## norm_methodmedianQC  0.06032    0.03525  9.01079   1.711 0.121169
## norm_methodPQN       0.02630    0.03525  9.01079   0.746 0.474606
## norm_methodquantile  0.02177    0.03525  9.01079   0.618 0.552166
## norm_methodSERRF    -0.18942    0.03525  9.01079  -5.374 0.000446 ***
## norm_methodTIC       0.02271    0.03525  9.01079   0.644 0.535511
## norm_methodTICQC     0.02271    0.03525  9.01079   0.644 0.535511
## ---
## Signif. codes:  0 '***' 0.001 '**' 0.01 '*' 0.05 '.' 0.1 ' ' 1
##
## Correlation of Fixed Effects:
##             (Intr) nrm_mthdl nrm_mthdlQC nrm_mthdm nrm_mthdmQC nr_PQN nrm_mthdq
## nrm_mthdlss -0.430
## nrm_mthdlQC -0.430  0.500
## nrm_mthdmdn -0.430  0.500     0.500
## nrm_mthdmQC -0.430  0.500     0.500       0.500
## nrm_mthdPQN -0.430  0.500     0.500       0.500     0.500
## nrm_mthdqnt -0.430  0.500     0.500       0.500     0.500       0.500
## nrm_mtSERRF -0.430  0.500     0.500       0.500     0.500       0.500  0.500
## nrm_mthdTIC -0.430  0.500     0.500       0.500     0.500       0.500  0.500
## nrm_mtTICQC -0.430  0.500     0.500       0.500     0.500       0.500  0.500
##             n_SERR nr_TIC
## nrm_mthdlss
## nrm_mthdlQC
## nrm_mthdmdn
## nrm_mthdmQC
## nrm_mthdPQN
## nrm_mthdqnt
## nrm_mtSERRF
## nrm_mthdTIC  0.500
## nrm_mtTICQC  0.500  0.500
```

```
# Linear mixed model fit by REML. t-tests use Satterthwaite's method [
# lmerModLmerTest]
# Formula: relative_dispersion ~ norm_method + (1 | dataset:Time) + (1 |
#     dataset:norm_method) + (1 | dataset:Trt)
#    Data: dispersion_compare_data
#
# REML criterion at convergence: -1107.7
#
# Scaled residuals:
#     Min      1Q  Median      3Q     Max
# -2.5235 -0.6214 -0.0929  0.4626  5.7832
#
# Random effects:
#  Groups              Name        Variance  Std.Dev.
#  dataset:norm_method (Intercept) 0.0009079 0.03013
#  dataset:Time        (Intercept) 0.0069668 0.08347
#  dataset:Trt         (Intercept) 0.0040380 0.06355
#  Residual                        0.0270979 0.16461
# Number of obs: 1620, groups:
```

```
# dataset:norm_method, 20; dataset:Time, 18; dataset:Trt, 6
#
# Fixed effects:
#                      Estimate Std. Error       df t value Pr(>|t|)
# (Intercept)           0.97080    0.04100 17.81983  23.676 6.47e-15 ***
# norm_methodloess      0.03169    0.03525  9.01079   0.899 0.391985
# norm_methodloessQC    0.02026    0.03525  9.01079   0.575 0.579502
# norm_methodmedian     0.06032    0.03525  9.01079   1.711 0.121169
# norm_methodmedianQC   0.06032    0.03525  9.01079   1.711 0.121169
# norm_methodPQN        0.02630    0.03525  9.01079   0.746 0.474606
# norm_methodquantile   0.02177    0.03525  9.01079   0.618 0.552166
# norm_methodSERRF     -0.18942    0.03525  9.01079  -5.374 0.000446 ***
# norm_methodTIC        0.02271    0.03525  9.01079   0.644 0.535511
# norm_methodTICQC      0.02271    0.03525  9.01079   0.644 0.535511
# ---
# Signif. codes:  0 '***' 0.001 '**' 0.01 '*' 0.05 '.' 0.1 ' ' 1

Cardio_metabolite.dispersion <- as.data.frame(summary(p5)$coefficients[,c("Estimate","Pr(>|t|)")]) %>% 
```

1. (Metabolomics:Cardio) Above model has decent homoscedasticity and normality.
2. (Metabolomics:Cardio) SERRF normalization showed significantly reduction in dispersion between replicates.

## Neuron

Read evaluation

```
# Neuron result
Eval_norm_method_RPpos <- readRDS("/Users/chiyen_tseng/Documents/Projects/Inception/metabolomics/Neurons
Eval_norm_method_RPneg <- readRDS("/Users/chiyen_tseng/Documents/Projects/Inception/metabolomics/Neurons
Eval_norm_method_Hilicpos <- readRDS("/Users/chiyen_tseng/Documents/Projects/Inception/metabolomics/Neu
# SERRF failed ; might due to very limited features in SERRF 1.7k vs 1k in SERRF normalized dataset; SE
Eval_norm_method_Hilicneg <- readRDS("/Users/chiyen_tseng/Documents/Projects/Inception/metabolomics/Neu
# SERRF failed ; might due to very limited features in SERRF 1.4k vs 0.5k in SERRF normalized dataset; 
```

Combine neuron metabolomics dispersion

```
# combine neuron metabiolomcis dispersion
RPpos.dis <- Eval_norm_method_RPpos$dispersion
RPneg.dis <- Eval_norm_method_RPneg$dispersion
HILICpos.dis <- Eval_norm_method_Hilicpos$dispersion
HILICneg.dis <- Eval_norm_method_Hilicneg$dispersion

Neuron_metabolomics_dis <- list(RPpos.dis, RPneg.dis, HILICpos.dis, HILICneg.dis)
Neuron_metabolomics_dis <- do.call("rbind",Neuron_metabolomics_dis)

dispersion_compare_data <- as.data.frame(Neuron_metabolomics_dis)
dispersion_compare_data$dataset <- as.factor(dispersion_compare_data$dataset)
dispersion_compare_data <- dispersion_compare_data %>% filter(Trt != "PooledQC") # remove pooled QC
```

Making linear mixed effect model with dataset, time, and Trt as random effect, and normalization method as fixed effect

```
p5 <- lmer(relative_dispersion ~ norm_method + (1 | dataset:Time) + (1 | dataset:norm_method) + (1 | da
          data = dispersion_compare_data)


# plot(p5) # residual plot
# qqnorm(resid(p5)) # QQ-normal plot
# summary(p5)
# norm_method relative_dispersion Time Trt          dataset
# 2844        SERRF           0.2931298  720 ACN Neuron_HILICneg
# 2862        SERRF           0.3048838  720 ACN Neuron_HILICneg
# 2094        SERRF           1.8500982  480  CP Neuron_HILICpos
# 2075        SERRF           2.0542457   30   C Neuron_HILICpos
# 2897        SERRF           2.2354915   60  CP Neuron_HILICneg

# These five SERRF normalized data casued extreme outlier in normal QQ plot

# Remove above data and refit model
p5 <- lmer(relative_dispersion ~ norm_method + (1 | dataset:Time) + (1 | dataset:norm_method) + (1 | da
          data = dispersion_compare_data[-c(2844,2862,2094,2075,2897),])


plot(p5) # residual plot
```

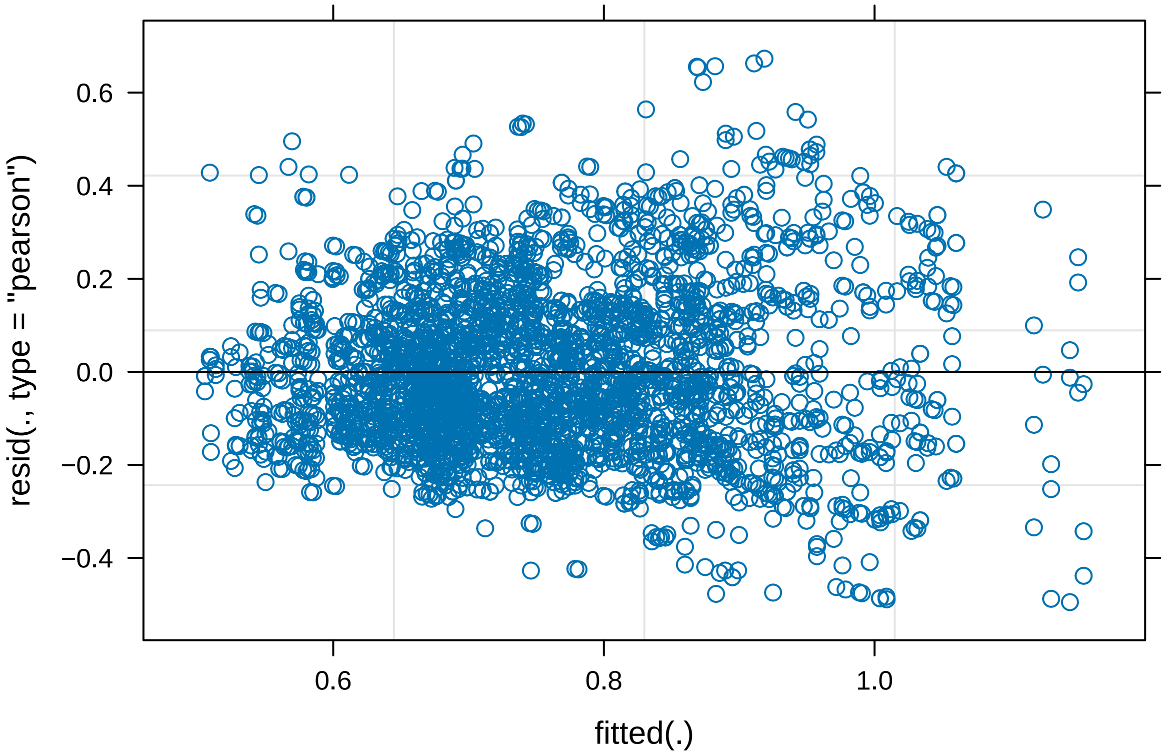


```
qqnorm(resid(p5)) # QQ-normal plot
```

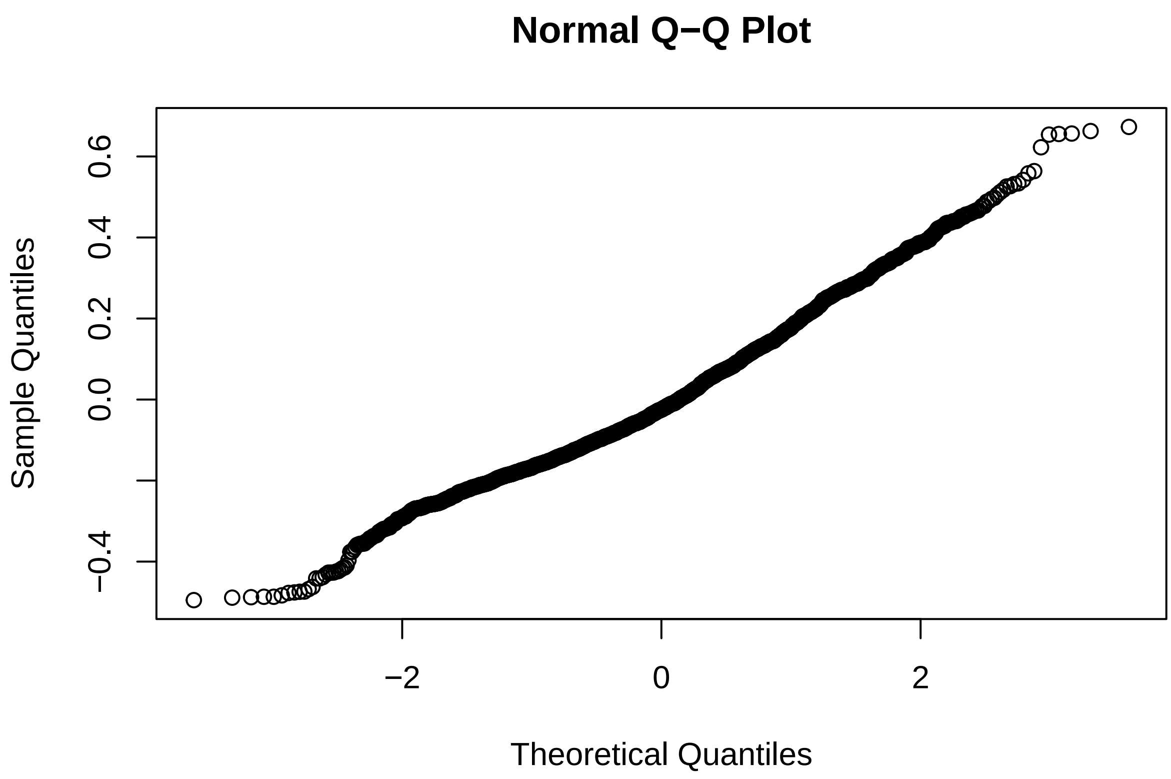


```r
summary(p5)
```

```
## Linear mixed model fit by REML. t-tests use Satterthwaite's method [
## lmerModLmerTest]
## Formula: relative_dispersion ~ norm_method + (1 | dataset:Time) + (1 |
##     dataset:norm_method) + (1 | dataset:Trt)
##    Data: dispersion_compare_data[-c(2844, 2862, 2094, 2075, 2897), ]
##
## REML criterion at convergence: -1760.4
##
## Scaled residuals:
##     Min      1Q  Median      3Q     Max
## -2.7978 -0.7085 -0.1389  0.6345  3.8025
##
## Random effects:
##  Groups              Name        Variance  Std.Dev.
##  dataset:norm_method (Intercept) 0.0047858 0.06918
##  dataset:Time        (Intercept) 0.0085124 0.09226
##  dataset:Trt         (Intercept) 0.0006597 0.02568
##  Residual                        0.0313137 0.17696
## Number of obs: 3235, groups:
## dataset:norm_method, 40; dataset:Time, 36; dataset:Trt, 12
##
## Fixed effects:
##                      Estimate Std. Error       df t value Pr(>|t|)
```

```
## (Intercept)          0.71342    0.03981 36.70833  17.922   <2e-16 ***
## norm_methodloess     0.05537    0.05085 24.28737   1.089    0.287
## norm_methodloessQC   0.04216    0.05085 24.28737   0.829    0.415
## norm_methodmedian    0.05808    0.05085 24.28737   1.142    0.265
## norm_methodmedianQC  0.05808    0.05085 24.28737   1.142    0.265
## norm_methodPQN       0.04801    0.05085 24.28737   0.944    0.354
## norm_methodquantile  0.03359    0.05085 24.28737   0.661    0.515
## norm_methodSERRF     0.08136    0.05087 24.31667   1.599    0.123
## norm_methodTIC       0.05069    0.05085 24.28737   0.997    0.329
## norm_methodTICQC     0.02308    0.05085 24.28737   0.454    0.654
## ---
## Signif. codes:  0 '***' 0.001 '**' 0.01 '*' 0.05 '.' 0.1 ' ' 1
##
## Correlation of Fixed Effects:
##             (Intr) nrm_mthdl nrm_mthdlQC nrm_mthdm nrm_mthdmQC nr_PQN nrm_mthdq
## nrm_mthdlss -0.639
## nrm_mthdlQC -0.639  0.500
## nrm_mthdmdn -0.639  0.500     0.500
## nrm_mthdmQC -0.639  0.500     0.500       0.500
## nrm_mthdPQN -0.639  0.500     0.500       0.500     0.500
## nrm_mthdqnt -0.639  0.500     0.500       0.500     0.500       0.500
## nrm_mtSERRF -0.639  0.500     0.500       0.500     0.500       0.500  0.500
## nrm_mthdTIC -0.639  0.500     0.500       0.500     0.500       0.500  0.500
## nrm_mtTICQC -0.639  0.500     0.500       0.500     0.500       0.500  0.500
##             n_SERR nr_TIC
## nrm_mthdlss
## nrm_mthdlQC
## nrm_mthdmdn
## nrm_mthdmQC
## nrm_mthdPQN
## nrm_mthdqnt
## nrm_mtSERRF
## nrm_mthdTIC  0.500
## nrm_mtTICQC  0.500  0.500
```

```
# Linear mixed model fit by REML. t-tests use Satterthwaite's method ['lmerModLmerTest']
# Formula: relative_dispersion ~ norm_method + (1 | dataset:Time) + (1 |      dataset:norm_method) + (1
#    Data: dispersion_compare_data[-c(2844, 2862, 2094, 2075, 2897), ]
#
# REML criterion at convergence: -1760.4
#
# Scaled residuals:
#     Min      1Q  Median      3Q     Max
# -2.7978 -0.7085 -0.1389  0.6345  3.8025
#
# Random effects:
#  Groups              Name        Variance  Std.Dev.
#  dataset:norm_method (Intercept) 0.0047858 0.06918
#  dataset:Time        (Intercept) 0.0085124 0.09226
#  dataset:Trt         (Intercept) 0.0006597 0.02568
#  Residual                        0.0313137 0.17696
# Number of obs: 3235, groups:  dataset:norm_method, 40; dataset:Time, 36; dataset:Trt, 12
#
# Fixed effects:
```

```
#                      Estimate Std. Error       df t value Pr(>|t|)
# (Intercept)           0.71342    0.03981 36.70833  17.922   <2e-16 ***
# norm_methodloess      0.05537    0.05085 24.28737   1.089    0.287
# norm_methodloessQC    0.04216    0.05085 24.28737   0.829    0.415
# norm_methodmedian     0.05808    0.05085 24.28737   1.142    0.265
# norm_methodmedianQC   0.05808    0.05085 24.28737   1.142    0.265
# norm_methodPQN        0.04801    0.05085 24.28737   0.944    0.354
# norm_methodquantile   0.03359    0.05085 24.28737   0.661    0.515
# norm_methodSERRF      0.08136    0.05087 24.31667   1.599    0.123
# norm_methodTIC        0.05069    0.05085 24.28737   0.997    0.329
# norm_methodTICQC      0.02308    0.05085 24.28737   0.454    0.654
# ---
# Signif. codes:  0 '***' 0.001 '**' 0.01 '*' 0.05 '.' 0.1 ' ' 1

Neuron_metabolite.dispersion <- as.data.frame(summary(p5)$coefficients[,c("Estimate","Pr(>|t|)")]) %>% 
```

1. (Metabolomics:Neuron) Above model has decent homoscedasticity and normality after removing a few SERRF normalized data points.

## Lipidomics

Read Lipidomics:Cardio evaluation metrics (only include positive mode)

```
# Cardio Lipidomics
Eval_norm_method_Lipidpos <- readRDS("/Users/chiyen_tseng/Documents/Projects/Inception/Lipidomics/Positi
# negative mode fail: too few MS/MS identified and too many below background,
pos <- Eval_norm_method_Lipidpos$eval %>% mutate(R2_trt_diff = (R2_trt/R2_trt[1]-1), R2_time_diff = (R2_
  pos$R2_trt_diff[pos$Adonis2_P_trt > 0.11] <- NA
  pos$R2_time_diff[pos$Adonis2_P_time > 0.11]  <- NA
  pos$R2_time_trt_diff[pos$Adonis2_P_time_trt > 0.11] <- NA
Cardio_lipidomics_eval <- pos
```

Combine cardio lipidomics dispersion

```
# combine cardio lipidomics dispersion
Lipidpos.dis <- Eval_norm_method_Lipidpos$dispersion

dispersion_compare_data <- as.data.frame(Lipidpos.dis)
dispersion_compare_data$dataset <- as.factor(dispersion_compare_data$dataset)
dispersion_compare_data <- dispersion_compare_data %>% filter(Trt != "PooledQC") %>% mutate(cell = "Card
cardio_dispersion_compare_data <- dispersion_compare_data
```

Making linear mixed effect model with time and Trt as random effect, and normalization method as fixed ef-

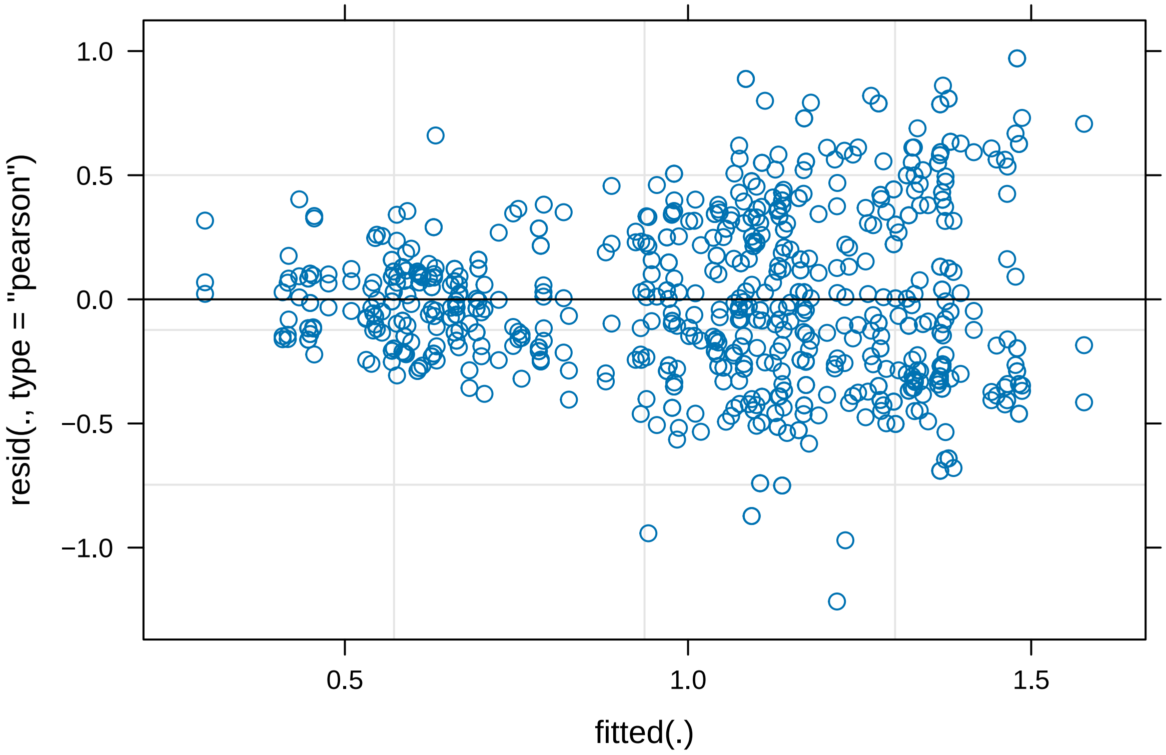


fect

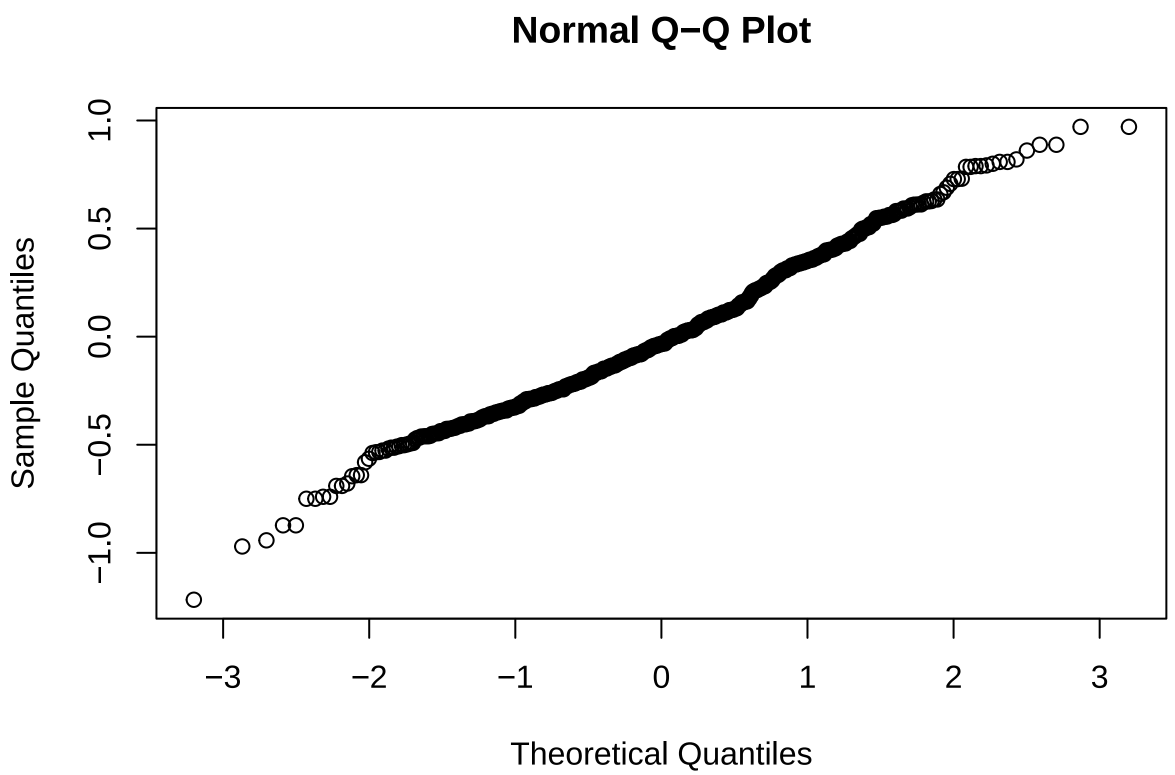


```
## Linear mixed model fit by REML. t-tests use Satterthwaite's method [
## lmerModLmerTest]
## Formula: relative_dispersion ~ norm_method + (1 | Time) + (1 | Time:Trt)
##    Data: dispersion_compare_data
##
## REML criterion at convergence: 605.5
##
## Scaled residuals:
##     Min      1Q  Median      3Q     Max
## -3.5909 -0.7175 -0.1000  0.6553  2.8638
##
## Random effects:
##  Groups   Name        Variance Std.Dev.
##  Time:Trt (Intercept) 0.07397  0.2720
##  Time     (Intercept) 0.02292  0.1514
##  Residual             0.11487  0.3389
## Number of obs: 729, groups:  Time:Trt, 27; Time, 9
##
## Fixed effects:
##                      Estimate Std. Error        df t value Pr(>|t|)
## (Intercept)           0.87761    0.08188  12.12613  10.718 1.53e-07 ***
## norm_methodloess      0.24798    0.05326 694.00005   4.656 3.86e-06 ***
## norm_methodloessQC    0.12056    0.05326 694.00005   2.264  0.02389 *
## norm_methodmedian     0.15320    0.05326 694.00005   2.877  0.00414 **
## norm_methodmedianQC   0.15320    0.05326 694.00005   2.877  0.00414 **
## norm_methodPQN        0.15758    0.05326 694.00005   2.959  0.00319 **
```

```
## norm_methodquantile   0.11304    0.05326 694.00005   2.123  0.03415 *
## norm_methodTIC        0.15045    0.05326 694.00005   2.825  0.00486 **
## norm_methodTICQC      0.15045    0.05326 694.00005   2.825  0.00486 **
## ---
## Signif. codes:  0 '***' 0.001 '**' 0.01 '*' 0.05 '.' 0.1 ' ' 1
##
## Correlation of Fixed Effects:
##             (Intr) nrm_mthdl nrm_mthdlQC nrm_mthdm nrm_mthdmQC nr_PQN nrm_mthdq
## nrm_mthdlss -0.325
## nrm_mthdlQC -0.325  0.500
## nrm_mthdmdn -0.325  0.500     0.500
## nrm_mthdmQC -0.325  0.500     0.500       0.500
## nrm_mthdPQN -0.325  0.500     0.500       0.500     0.500
## nrm_mthdqnt -0.325  0.500     0.500       0.500     0.500       0.500
## nrm_mthdTIC -0.325  0.500     0.500       0.500     0.500       0.500  0.500
## nrm_mtTICQC -0.325  0.500     0.500       0.500     0.500       0.500  0.500
##             nr_TIC
## nrm_mthdlss
## nrm_mthdlQC
## nrm_mthdmdn
## nrm_mthdmQC
## nrm_mthdPQN
## nrm_mthdqnt
## nrm_mthdTIC
## nrm_mtTICQC  0.500
```

1. (Lipidomics:Cardio) Above model has decent normality but not homoscedasticity
2. (Lipidomics:Cardio) Most normalization showed significantly increase in dispersion between replicates after normalization.

### Neuron

Read Neuron:Lipidomics evaluation metrics

```
# Neuron Lipidomics
Eval_norm_method_Lipidpos <- readRDS("/Users/chiyen_tseng/Documents/Projects/Inception/Lipidomics/Positi
Eval_norm_method_Lipidneg <- readRDS("/Users/chiyen_tseng/Documents/Projects/Inception/Lipidomics/Negati
```

Combine pos and neg mode dispersion

- Making linear mixed effect model with time and Trt as random effect, and normalization method as

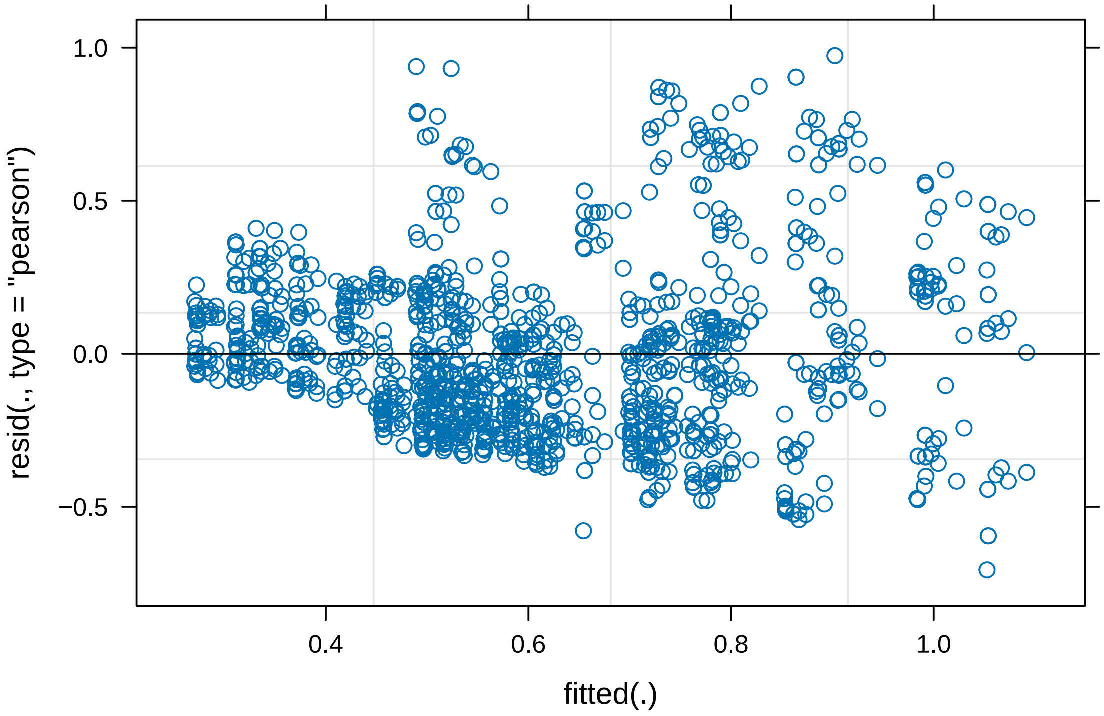


fixed effect

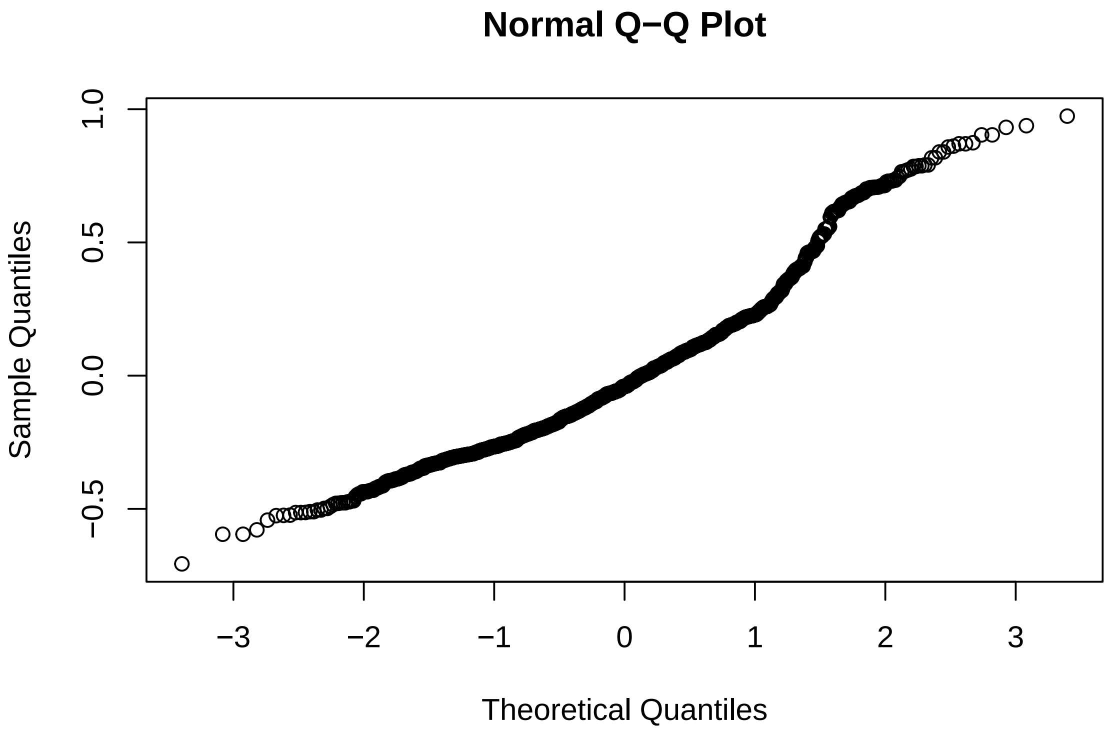


```
## Linear mixed model fit by REML. t-tests use Satterthwaite's method [
```

```
## lmerModLmerTest]
## Formula: relative_dispersion ~ norm_method + (1 | dataset:Time) + (1 |
##     dataset:Trt)
##    Data: dispersion_compare_data
##
## REML criterion at convergence: 579.2
##
## Scaled residuals:
##     Min      1Q  Median      3Q     Max
## -2.4860 -0.7216 -0.1411  0.5048  3.4284
##
## Random effects:
##  Groups       Name        Variance Std.Dev.
##  dataset:Time (Intercept) 0.01716  0.1310
##  dataset:Trt  (Intercept) 0.02612  0.1616
##  Residual                 0.08070  0.2841
## Number of obs: 1458, groups:  dataset:Time, 18; dataset:Trt, 6
##
## Fixed effects:
##                      Estimate Std. Error        df t value Pr(>|t|)
## (Intercept)         6.005e-01  7.619e-02 8.209e+00   7.882 4.21e-05 ***
## norm_methodloess    3.918e-02  3.156e-02 1.428e+03   1.241    0.215
## norm_methodloessQC  1.416e-02  3.156e-02 1.428e+03   0.449    0.654
## norm_methodmedian   8.360e-04  3.156e-02 1.428e+03   0.026    0.979
## norm_methodmedianQC 8.360e-04  3.156e-02 1.428e+03   0.026    0.979
## norm_methodPQN      2.097e-02  3.156e-02 1.428e+03   0.664    0.507
## norm_methodquantile 8.876e-03  3.156e-02 1.428e+03   0.281    0.779
## norm_methodTIC      1.218e-03  3.156e-02 1.428e+03   0.039    0.969
## norm_methodTICQC    1.218e-03  3.156e-02 1.428e+03   0.039    0.969
## ---
## Signif. codes:  0 '***' 0.001 '**' 0.01 '*' 0.05 '.' 0.1 ' ' 1
##
## Correlation of Fixed Effects:
##             (Intr) nrm_mthdl nrm_mthdlQC nrm_mthdm nrm_mthdmQC nr_PQN nrm_mthdq
## nrm_mthdlss -0.207
## nrm_mthdlQC -0.207  0.500
## nrm_mthdmdn -0.207  0.500     0.500
## nrm_mthdmQC -0.207  0.500     0.500       0.500
## nrm_mthdPQN -0.207  0.500     0.500       0.500     0.500
## nrm_mthdqnt -0.207  0.500     0.500       0.500     0.500       0.500
## nrm_mthdTIC -0.207  0.500     0.500       0.500     0.500       0.500  0.500
## nrm_mtTICQC -0.207  0.500     0.500       0.500     0.500       0.500  0.500
##             nr_TIC
## nrm_mthdlss
## nrm_mthdlQC
## nrm_mthdmdn
## nrm_mthdmQC
## nrm_mthdPQN
## nrm_mthdqnt
## nrm_mthdTIC
## nrm_mtTICQC  0.500
```

1. (Lipidomics:Neuron) Normality: decent until higher dispersion; homoscedasticity: slightly increase variation

2. (Lipidomics:Neuron) None of normalization methods showed significantly increase in dispersion between replicates after normalization.

## Proteomics

Read data, both Cardio and Neuron

```
# Proteomics
Eval_norm_method_CardioProteomics <- readRDS("/Users/chiyen_tseng/Documents/Projects/Inception/proteomi
Cardio_proteomics_eval <- Eval_norm_method_CardioProteomics$eval %>% mutate(R2_trt_diff = (R2_trt/R2_tr

Eval_norm_method_NeuronProteomics <- readRDS("/Users/chiyen_tseng/Documents/Projects/Inception/proteomi
Neuron_proteomics_eval <- Eval_norm_method_NeuronProteomics$eval %>% mutate(R2_trt_diff = (R2_trt/R2_tr
```

Combine dispersion

```
# combine neuron proteomics dispersion
Cardioproteomics.dis <- Eval_norm_method_CardioProteomics$dispersion
  dispersion_compare_data <- as.data.frame(Cardioproteomics.dis)
  dispersion_compare_data$dataset <- as.factor(dispersion_compare_data$dataset)
  dispersion_compare_data <- dispersion_compare_data %>% filter(Trt != "PooledQC") # remove Pooled QC
  cardio_dispersion_compare_data <- dispersion_compare_data
```

Making linear mixed effect model with time and Trt as random effect, and normalization method as fixed effect

```
p4 <- lmer(relative_dispersion ~ norm_method + (1 | dataset:Time)  + (1 | dataset:Trt) ,
             data = dispersion_compare_data)
  plot(p4)
```

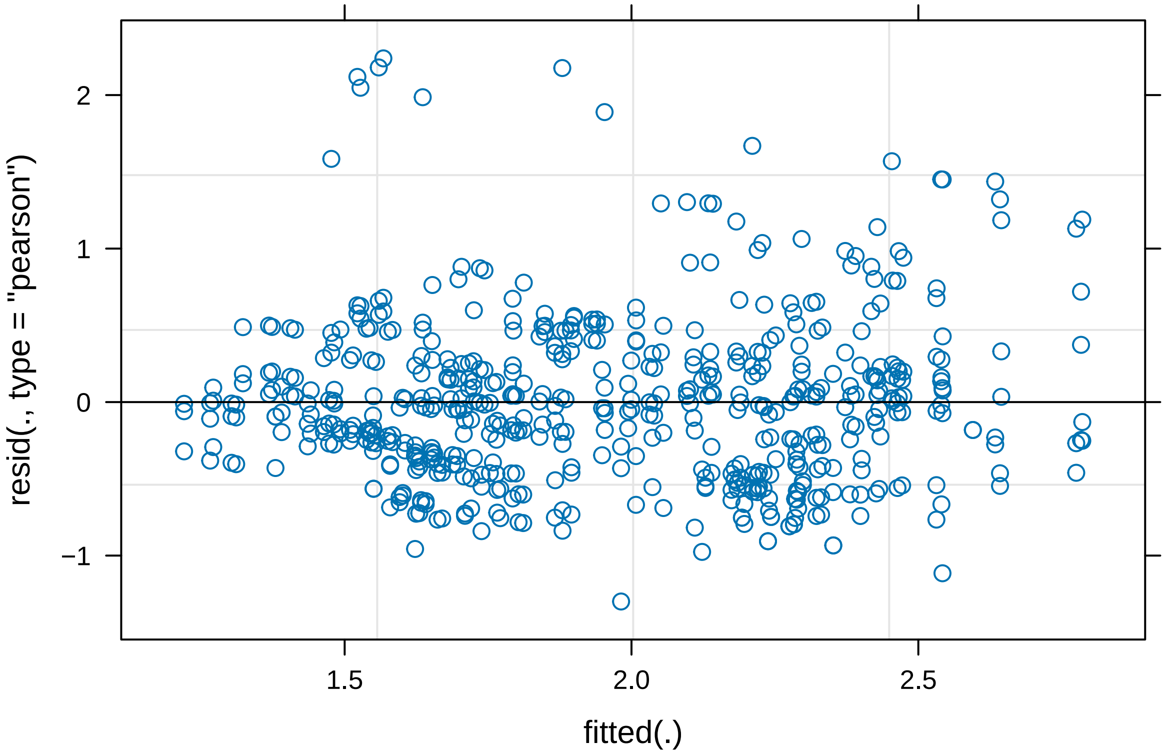


```
qqnorm(resid(p4))
```

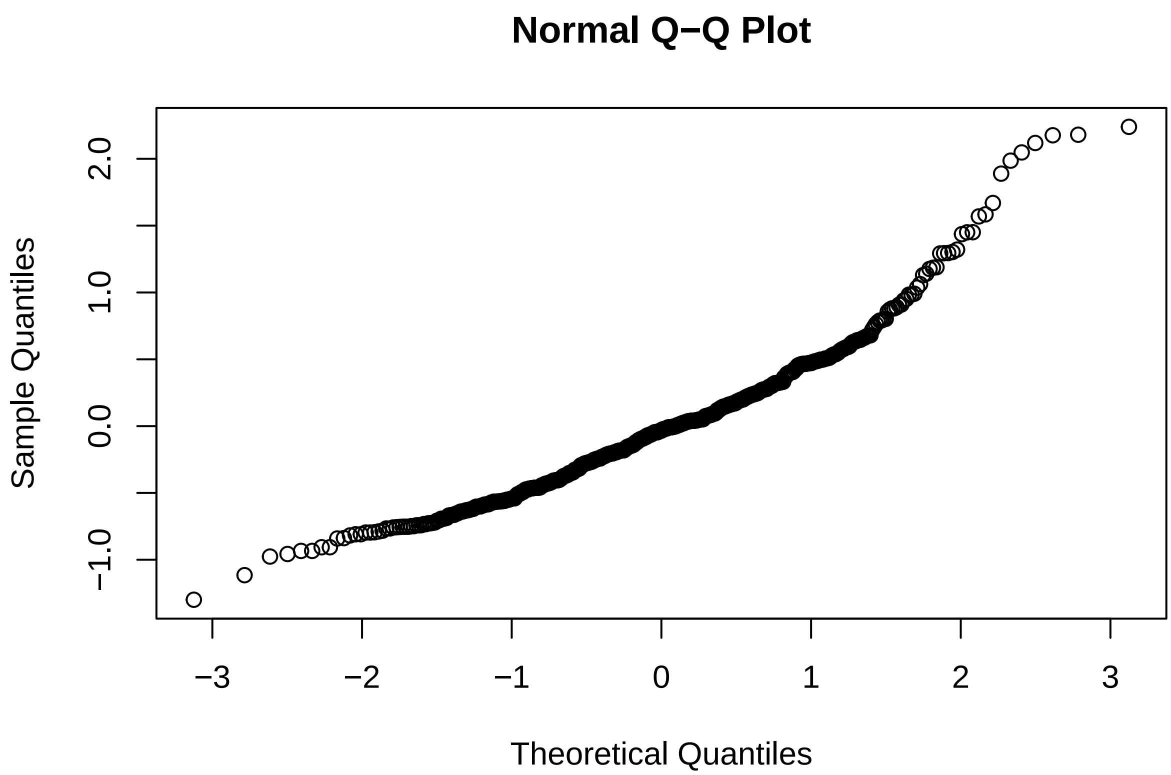


```
Cardio_protein.dispersion <- as.data.frame(summary(p4)$coefficients[,c("Estimate","Pr(>|t|)")]) %>% rown


#   Linear mixed model fit by REML. t-tests use Satterthwaite's method [
# lmerModLmerTest]
# Formula: relative_dispersion ~ norm_method + (1 | dataset:Time) + (1 |
#     dataset:Trt)
#    Data: dispersion_compare_data
#
# REML criterion at convergence: 966.7
#
# Scaled residuals:
#     Min      1Q  Median      3Q     Max
# -2.3703 -0.7184 -0.0601  0.4940  4.0852
#
# Random effects:
#  Groups       Name        Variance Std.Dev.
#  dataset:Time (Intercept) 0.13314  0.3649
#  dataset:Trt  (Intercept) 0.01644  0.1282
#  Residual                 0.30044  0.5481
# Number of obs: 560, groups:  dataset:Time, 9; dataset:Trt, 3
#
# Fixed effects:
#                       Estimate Std. Error        df t value Pr(>|t|)
# (Intercept)            2.25102    0.15501  12.13866  14.521 4.88e-09 ***
# norm_methodloess      -0.35181    0.08667 542.99104  -4.059 5.64e-05 ***
```

```
# norm_methodmedian    -0.31990    0.08667 542.99104  -3.691 0.000246 ***
# norm_methodPQN       -0.31186    0.08667 542.99104  -3.598 0.000350 ***
# norm_methodquantile  -0.35715    0.08667 542.99104  -4.121 4.36e-05 ***
# norm_methodTIC       -0.24332    0.08667 542.99104  -2.808 0.005172 **
# norm_methodVSN       -0.40262    0.08667 542.99104  -4.646 4.26e-06 ***
# ---
# Signif. codes:  0 '***' 0.001 '**' 0.01 '*' 0.05 '.' 0.1 ' ' 1
#
# Correlation of Fixed Effects:
#             (Intr) nrm_mthdl nrm_mthdm nr_PQN nrm_mthdq nr_TIC
# nrm_mthdlss -0.280
# nrm_mthdmdn -0.280  0.500
# nrm_mthdPQN -0.280  0.500     0.500
# nrm_mthdqnt -0.280  0.500     0.500     0.500
# nrm_mthdTIC -0.280  0.500     0.500     0.500  0.500
# nrm_mthdVSN -0.280  0.500     0.500     0.500  0.500     0.500
```

1. (Proteomics:Cardio) Normality: decent until higher dispersion; homoscedasticity: decent
2. (Proteomics:Cardio) All normalization methods showed significantly decrease in dispersion between replicates after normalization.

## Neuron

```
## Linear mixed model fit by REML. t-tests use Satterthwaite's method [
## lmerModLmerTest]
## Formula: relative_dispersion ~ norm_method + (1 | dataset:Time) + (1 |
##     dataset:Trt)
##    Data: dispersion_compare_data
##
## REML criterion at convergence: 371.6
##
## Scaled residuals:
##     Min      1Q  Median      3Q     Max
## -1.8902 -0.6616 -0.1252  0.5893  3.6184
##
## Random effects:
##  Groups       Name        Variance Std.Dev.
##  dataset:Time (Intercept) 0.02392  0.1546
##  dataset:Trt  (Intercept) 0.01680  0.1296
##  Residual                 0.10218  0.3197
## Number of obs: 567, groups:  dataset:Time, 9; dataset:Trt, 3
##
## Fixed effects:
##                      Estimate Std. Error         df t value Pr(>|t|)
## (Intercept)          0.926194   0.097557   5.116311   9.494 0.000193 ***
## norm_methodloess    -0.063227   0.050229 549.999790  -1.259 0.208652
## norm_methodmedian   -0.002983   0.050229 549.999790  -0.059 0.952671
## norm_methodPQN      -0.011302   0.050229 549.999790  -0.225 0.822063
## norm_methodquantile  0.024417   0.050229 549.999790   0.486 0.627089
## norm_methodTIC       0.012238   0.050229 549.999790   0.244 0.807605
## norm_methodVSN       0.065955   0.050229 549.999790   1.313 0.189704
## ---
```

```
## Signif. codes:  0 '***' 0.001 '**' 0.01 '*' 0.05 '.' 0.1 ' ' 1
##
## Correlation of Fixed Effects:
##              (Intr) nrm_mthdl nrm_mthdm nr_PQN nrm_mthdq nr_TIC
## nrm_mthdlss -0.257
## nrm_mthdmdn -0.257  0.500
## nrm_mthdPQN -0.257  0.500     0.500
## nrm_mthdqnt -0.257  0.500     0.500     0.500
## nrm_mthdTIC -0.257  0.500     0.500     0.500  0.500
## nrm_mthdVSN -0.257  0.500     0.500     0.500  0.500     0.500
```

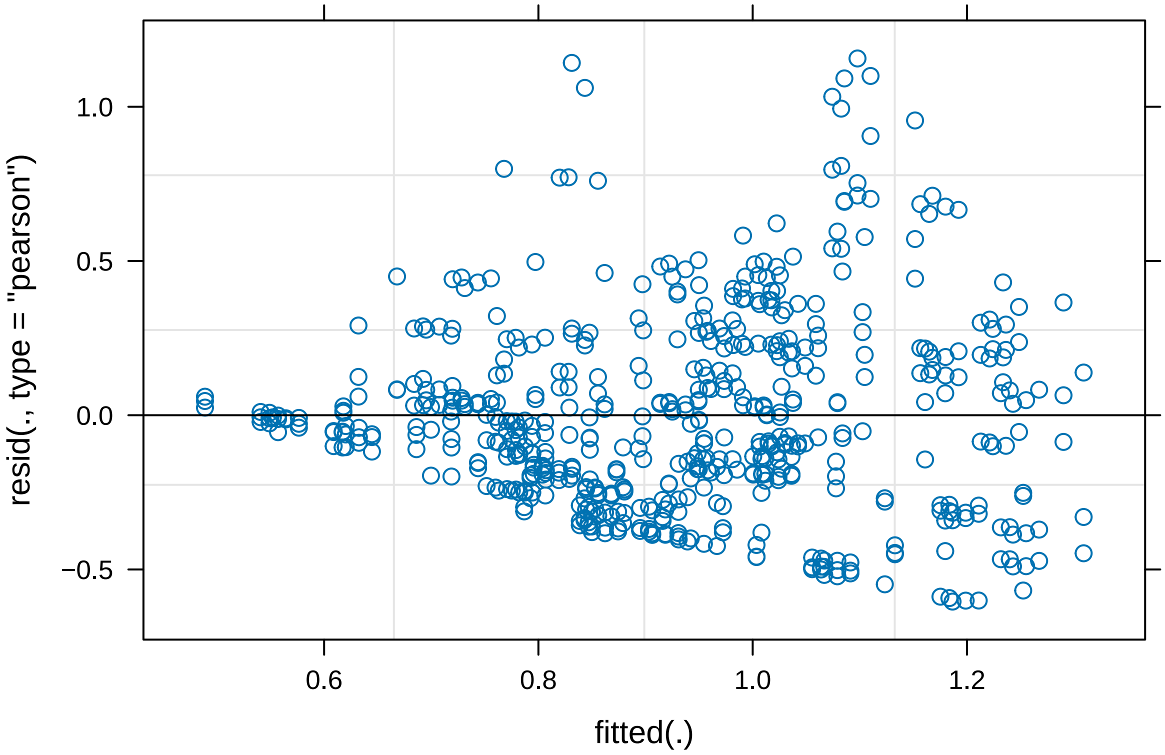

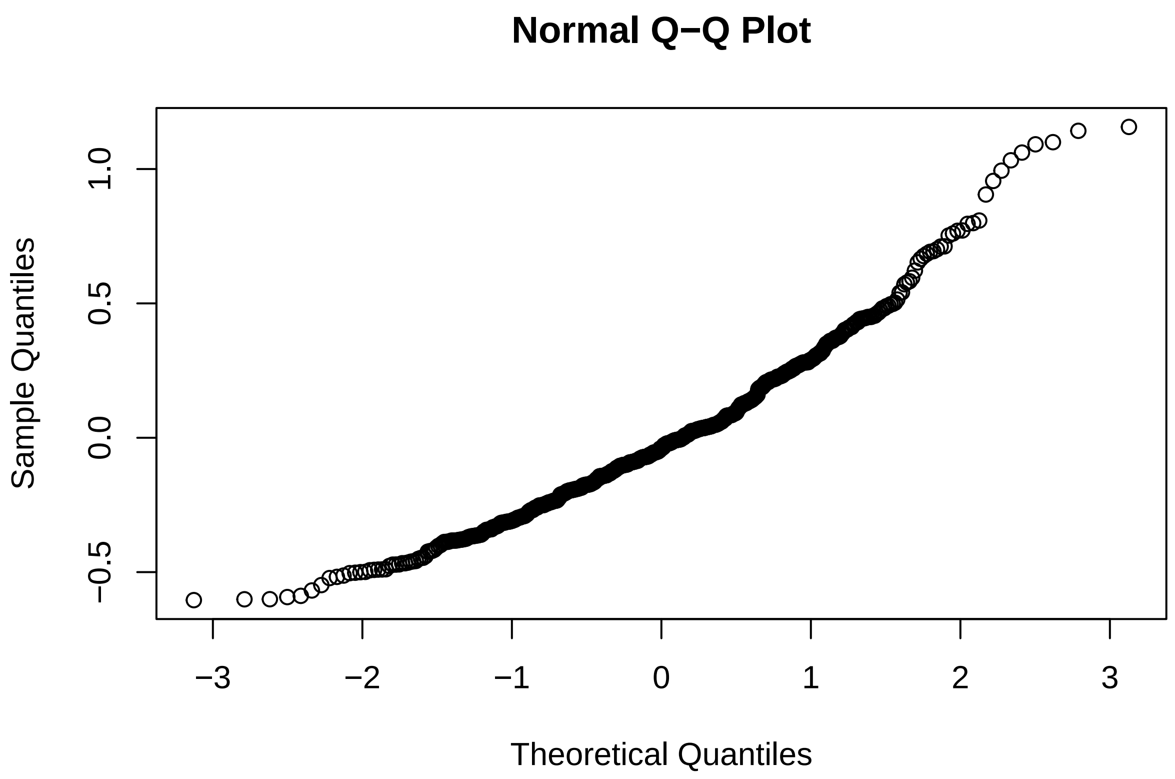


1. (Proteomics:Neuron) Normality: decent ; homoscedasticity: increae variation with dispersion 2. (Proteomics:Neuron) No normalization methods showed significantly change in dispersion between replicates after normalization.

**Output**

```
All_dispersion_testing_after_norm.list <- apropos(".dispersion$")
All_dispersion_testing_after_norm <- lapply(1:6, function(x) get(All_dispersion_testing_after_norm.list
All_dispersion_testing_after_norm <- do.call("rbind",All_dispersion_testing_after_norm)

# write_csv(All_dispersion_testing_after_norm , "Norm_paper_draft1/supplementrary/S4_table.csv")
```
