# Supplementary material for: Evaluation of normalization strategies for mass spectrometry-based multi-omics datasets
Source: Metabolomics. 2025 Jul 1;21(4):98. doi: 10.1007/s11306-025-02297-1 (PMC12214035; doi:10.1007/s11306-025-02297-1)
Supplement: Supplementary file 4 — Supplementary material 4 (PDF 229.4 kb) [file 11306_2025_2297_MOESM4_ESM.pdf]

Supplementary Script

Chi Yen Tseng

March 24, 2025

Normalization script

Load limma, vsn, affy, vegan, permute,mgcv R packages

```
library(limma)
library(vsn)
library(affy)
library(vegan)
library(permute)
library(mgcv)
library(tidyverse)
```

data `dim(data) = [1] 1249   98`

Normalization Methods QC-independent: Median, Quantile, LOESS, Total Intensity (TIC), VSN

```
# Median normalization
data.median <- normalizeBetweenArrays(data, method = "scale")

# Quantile normalization
data.quantile <- normalizeBetweenArrays(data, method = "quantile")

# LOESS normalization
data.loess <- 2^(normalizeBetweenArrays(log2(data), method = "cyclicloess"))

# TIC normalization
global.mean.ratio <- colSums(data)/mean(colSums(data))
# calculate total feature intensity for each sample relative to average total
#feature intensity across samples
data.TIC <- t(t(df.samp.mat)/global.mean.ratio) %>% as.data.frame()
# Adjust individual feature intensity

# VSN normalization
data.vsn <- justvsn(as.matrix(data))
```

QC-dependent: PQN, MedianQC, LOESSQC, TICQC, SERRF(directly acquired from Compound Discover)

```
# PQN normalization (run after TIC)
Ref_spectrum <- apply(data.TIC[,grep("PooledQC",colnames(data.TIC))],1,median)
# define Reference spectrum as the median intensity across all pooled QC samples
global.relative.ratio <- apply(data.TIC,2, FUN=function(x) x/Ref_spectrum)
```

```
# adjust individual feature intensity relative to Ref_spectrum
global.relative.ratio.median <- apply(global.relative.ratio,2,median)
# global (across all features) adjusting factor for each sample is determined
#by median of feature adjusting factor (global.relative.ratio)
data.PQN <- sapply(1:ncol(data.TIC), function(x)
  data.TIC[,x]/global.relative.ratio.median[x])
# each sample feature intensity was corrected for global (across all features) adjusting factor
# <NOTE> for proteomics data, there is no pooled QC samples, we used all the samples to build Ref_spect

# MedianQC normalization
# define QC sample location
QC_loc <- grep("PooledQC", colnames(data))
# Median feature intensity from only pooled-QC samples
QC_median <- median(apply(data[,QC_loc], 2, median))
# Make correction factor
correction_factor <- QC_median/apply(data, 2, median)
data.medianQC <- t(t(data)*correction_factor)

# LOESSQC normalization
# nearliest QC sample (median of nearest 3 QC samples)
sink("temp.txt") # Could take a long time
data.loessQC <- {}
for (i in 1:ncol(data)) {
norm_QC_loess.sub <- normalize.loess(data[,unique(c(i,QC_loc))],
                                      subset = 1:nrow(data), log.it = TRUE)
# Run cyclic LOESS by select one sample and all pooled Samples, turn on log transformation
data.loessQC <- cbind(data.loessQC,norm_QC_loess.sub[,1])
}
colnames(data.loessQC) <- colnames(data)
sink()

# TICQC normalization
column.QConly <- grep("PooledQC", colnames(data)) # pooled QC column location
data.QConly <- data[,c(column.QConly)] # grep only pooled QC samples
global.mean.ratio <- colSums(data)/mean(colSums(data.QConly))
# adjusting factor is determined only from pooled QC samples; total intensity
# of each samples relative the average total intensity across pooled QC samples
data.TICQC <- t(t(data)/global.mean.ratio) %>% as.data.frame()
```

Evaluation of normalization effectiveness

1: Determine significantly differentiated features

```
# Run Gam model
result <- {} # differentiated result
result.name <- {} # differentiated feature
for (feature in TestFeatures) {
mod_gam = gam(intensity ~ Treatment + s(Exposure_Time, k = 3, by=Treatment) +
              s(Exposure_Time, k=3), data = GAM.data %>% filter(ID == feature), method = "REML")
# individual feature intensity as response variable, Treatment as fixed effect,
# Exposure time points and Exposure, Treatment interaction as smoothing effect,
```

```
# evaluated by Restricted Maximum Likelihood (REML); knot(k) (3 to 5) gives similar results
sig.trt <- summary(mod_gam)$p.table
if (sum(sig.trt[,4][-1] < 0.05) > 0) {
result <- rbind(result, sig.trt[,4][-1])
result.name <- c(result.name, feature)
}
}
result <- as_tibble(result)
result <- result %>% mutate(ID = result.name)
result <- result %>% mutate(A.padj = p.adjust(TreatmentA, method= "fdr",
          n = length(TestFeatures)), B.padj = p.adjust(TreatmentB, method= "fdr"),
          n = length(TestFeatures))
# significantly differentiated features between any treatments were determined
# (p < 0.05), and FDR was also calculated using default Benjamini & Hochberg (1995)
```

2: Estimate the variance explained by Time or Treatment (PERMANOVA)

- adonis2(PERMANOVA): PERMANOVA compares the variation between groups to the variation within groups.The test statistic, pseudo-F, is modeled after the F-statistic from ANOVA.
- anosim: ANOSIM is a non-parametric test based on the rank distances among sample units. If a grouping variable is important, the mean rank distance among sample units within a group will be smaller than the rank distance between sample units from different groups.

```
permutations = 10000
numcores = 4
set.seed(111)

# scale data, first column is feature ID
data.standardlize <- data[,2:ncol(data)]/rowSums(data[,2:ncol(data)])
data.standardlize <- data.standardlize %>% mutate(ID = data$ID, .before = 1)
# use scaled data for now
# data wangling to long format
data.standardlize <- data.standardlize %>% gather(key = "sample",
                                value = "intensity", 2:ncol(data.standardlize))
data.standardlize <- data.standardlize %>% left_join(inception_sample_Naming_map,
                            by = join_by(sample == `File Name`)) # Add meta data
data.standardlize$Treatment <- as.factor(data.standardlize$Treatment)
data.standardlize$Treatment <- relevel(data.standardlize$Treatment, ref = "ACN")
                                                  # use ACN as reference level

data.df <- spread(data.standardlize[,c("sample", "ID","intensity")],
  key= ID, value = intensity) # select sample, ID, feature intensity as wide format
data.df1 <- data.df[,2:ncol(data.df)]

# Make meta data,including Treatment and Exposure_Time
data.meta <- as_tibble(data.standardlize)
data.meta <- data.meta %>% select(sample, Treatment, Exposure_Time) %>% distinct()
data.meta <- data.meta[match(data.df$sample,data.meta$sample) ,]
data.meta <- data.meta %>% mutate(Treat_Time = paste0(data.meta$Treatment,"_",
                                                      data.meta$Exposure_Time))
data.meta$Exposure_Time <- as.factor(data.meta$Exposure_Time)
data.meta$Treat_Time <- as.factor(data.meta$Treat_Time)
```

```
# Run adonis2 (PERMANOVA) to determine variance explained by Treatment,
# Exposure_Time, and their interaction. Using Bray-Curtis dissimilarity non-parametric statistics
group_adonis <- adonis2(GAM.dataframe1 ~ Treatment*Exposure_Time,
                data = GAM.meta,
                dist = "bray",
                permutations = permutations,
                by = "term", parallel = numcores, method = "bray")

print(group_adonis)

# Run anosim to make sure the grouping is real becasue adonis2 can only be used
# to determine the separation of the centroid
group_anosim<- anosim(as.matrix(data.df), grouping = as.factor(data.meta$Exposure_Time), permutations =
                , parallel = numcores)
print(group_anosim)

# Variance explained by Treatment
c(group_adonis$`Pr(>F)`[1],group_adonis$R2[1]) # significance and variance
                                               # explained by Treatment

# Variance explained by Time
c(group_adonis$`Pr(>F)`[2],group_adonis$R2[2]) # significance and variance explained by Time

# group separation: anosim
group_anosim$signif
```

3: Estimate the dispersion between bio-replicates and pooled-QC samples using vegdist, R

```
# Calculate dispersion using all data
dis.data <- data
# scale the GAM data
dis.data.standardlize <- dis.data[,2:ncol(dis.data)]/rowSums(dis.data[,2:ncol(dis.data)])
dis.data.standardlize <- dis.data.standardlize %>% mutate(ID = dis.data$ID, .before = 1)
# use scaled data for now
dis.data <- dis.data.standardlize
# data wangling
dis.data <- dis.data %>% gather(key = "sample", value = "intensity", 2:ncol(dis.data))
dis.data <- dis.data %>% left_join(inception_sample_Naming_map, by = join_by(sample == `File Name`))
colnames(dis.data)[colnames(dis.data) == "Exposure Time"] <- "Exposure_Time"
dis.data$`Batch #` <- as.factor(dis.data$`Batch #`)
dis.dataframe <- spread(dis.data[,c("sample", "ID","intensity")], key= ID, value = intensity)
dis.dataframe1 <- dis.dataframe
# make meta data
dis.meta <- as_tibble(dis.data)
dis.meta <- dis.meta %>% select(sample, Treatment, Exposure_Time) %>% distinct()
# make sure it matches order
dis.meta <- dis.meta[match(dis.dataframe$sample,dis.meta$sample) ,]
dis.meta <- dis.meta %>% mutate(Treat_Time = paste0(dis.meta$Treatment,"_",dis.meta$Exposure_Time))
dis.meta$Treat_Time[grep("_PooledQC_",dis.meta$sample)] <- "PooledQC"
dis.meta$Treatment[grep("_PooledQC_",dis.meta$sample)] <- "PooledQC"
dis.meta$Exposure_Time[grep("_PooledQC_",dis.meta$sample)] <- "PooledQC"
```

```
# calculate dispersion between bio-replicate; Using PERMDISP for the analysis
# of multivariate homogeneity of group dispersions (variances)
dis <- vegdist(as.matrix(dis.dataframe1[,-1])) # distance measure using
                                                # Bray-Curtis between each sample
perm_betadisper <- betadisper(d = dis, group = as.factor(dis.meta$Treat_Time), type = "centroid")
# calculate homogeneity of group dispersions relative to group centroid,
# the group here is defined as the bio-replicates with the same treatment and collected at the same tim
print(perm_betadisper$distances)

# calculate relative dispersion between bio-replicate against Media control
dispersion_treatment <- tibble(dispersion = perm_betadisper$distances,
                               Trt = dis.meta$Treatment, Time = dis.meta$Exposure_Time)
average_dispersion_MED <- dispersion_treatment %>% filter(Trt == "MED") %>%
  summarise(MED_disp = exp(mean(log(dispersion)))) %>% pull(MED_disp)
  # calculate group dispersions at media control
dispersion_treatment <- dispersion_treatment %>%
  mutate(dispersion_cor = dispersion/average_dispersion_MED)
  # calculate relative group dispersions to media control
dispersion_treatment
```

4: Estimate the feature consistency based on pooled QC RSD (number of feature with RSD < 0.2 and median RSD after normalization)

```
RSD.data <- data
# Removing media control
RSD.data <- RSD.data[,-grep("_0_MED",colnames(RSD.data))]
# select pooled QC only
RSD.data.qc <- RSD.data[,grep("PooledQC",colnames(RSD.data))]
# relative RSD
rsd <- {}
for (i in 1:length(RSD.data$ID)) {
rsd_temp <- sd(as.numeric(GAM.data.qc[i,-1]))/mean(as.numeric(RSD.data.qc[i,-1]))
# rsd = sd(Xi) / mean(Xi)
rsd <- c(rsd, rsd_temp)
}

# number of feature with RSD < 0.2
sum(rsd < 0.2)

# median RSD after normalization
median(rsd)
```

```
sessionInfo()
```

```
## R version 4.4.1 (2024-06-14)
## Platform: aarch64-apple-darwin20
## Running under: macOS Ventura 13.7.4
##
## Matrix products: default
## BLAS:   /Library/Frameworks/R.framework/Versions/4.4-arm64/Resources/lib/libRblas.0.dylib
## LAPACK: /Library/Frameworks/R.framework/Versions/4.4-arm64/Resources/lib/libRlapack.dylib;  LAPACK v
```

```
##
## locale:
## [1] en_US.UTF-8/en_US.UTF-8/en_US.UTF-8/C/en_US.UTF-8/en_US.UTF-8
##
## time zone: America/Denver
## tzcode source: internal
##
## attached base packages:
## [1] stats     graphics  grDevices utils     datasets  methods   base
##
## other attached packages:
##  [1] lubridate_1.9.3     forcats_1.0.0       stringr_1.5.1
##  [4] dplyr_1.1.4         purrr_1.0.2         readr_2.1.5
##  [7] tidyr_1.3.1         tibble_3.2.1        ggplot2_3.5.1
## [10] tidyverse_2.0.0     mgcv_1.9-1          nlme_3.1-166
## [13] vegan_2.6-8         lattice_0.22-6      permute_0.9-7
## [16] affy_1.82.0         vsn_3.72.0          Biobase_2.64.0
## [19] BiocGenerics_0.50.0 limma_3.60.6
##
## loaded via a namespace (and not attached):
##  [1] utf8_1.2.4            generics_0.1.3        stringi_1.8.4
##  [4] hms_1.1.3             digest_0.6.37         magrittr_2.0.3
##  [7] timechange_0.3.0      evaluate_1.0.0        grid_4.4.1
## [10] fastmap_1.2.0         Matrix_1.7-0          BiocManager_1.30.25
## [13] fansi_1.0.6           scales_1.3.0          preprocessCore_1.66.0
## [16] cli_3.6.3             rlang_1.1.4           munsell_0.5.1
## [19] splines_4.4.1         withr_3.0.1           yaml_2.3.10
## [22] tools_4.4.1           parallel_4.4.1        tzdb_0.4.0
## [25] colorspace_2.1-1      vctrs_0.6.5           R6_2.5.1
## [28] lifecycle_1.0.4       zlibbioc_1.50.0       MASS_7.3-61
## [31] cluster_2.1.6         pkgconfig_2.0.3       affyio_1.74.0
## [34] pillar_1.9.0          gtable_0.3.5          glue_1.8.0
## [37] statmod_1.5.0         xfun_0.48             tidyselect_1.2.1
## [40] rstudioapi_0.16.0     knitr_1.48            htmltools_0.5.8.1
## [43] rmarkdown_2.28        compiler_4.4.1
```
